# Available Therapeutic Options for Corneal Neovascularization: A Review

**DOI:** 10.3390/ijms25105479

**Published:** 2024-05-17

**Authors:** Łukasz Drzyzga, Dorota Śpiewak, Mariola Dorecka, Dorota Wyględowska-Promieńska

**Affiliations:** 1 Department of Ophthalmology, Prof. K. Gibiński University Clinical Center, Medical University of Silesia, 40-055 Katowice, Poland; 2 Clinical Ophthalmology Center Okolux, 40-754 Katowice, Poland; 3 Department of Ophthalmology, Faculty of Medical Sciences in Katowice, Medical University of Silesia, 40-514 Katowice, Poland

**Keywords:** cornea, neovascularization, angiogenic factors, antiangiogenic factors, steroids, nonsteroidal anti-inflammatory drugs, anti-VEGF drugs, immunosuppressive drugs, fine needle diathermy, amniotic membrane transplantation, keratoplasty

## Abstract

Corneal neovascularization can impair vision and result in a poor quality of life. The pathogenesis involves a complex interplay of angiogenic factors, notably vascular endothelial growth factor (VEGF). This review provides a comprehensive overview of potential therapies for corneal neovascularization, covering tissue inhibitors of metalloproteinases (TIMPs), transforming growth factor beta (TGF-β) inhibitors, interleukin-1L receptor antagonist (IL-1 Ra), nitric oxide synthase (NOS) isoforms, galectin-3 inhibitors, retinal pigment epithelium-derived factor (PEDF), platelet-derived growth factor (PDGF) receptor inhibitors, and surgical treatments. Conventional treatments include anti-VEGF therapy and laser interventions, while emerging therapies such as immunosuppressive drugs (cyclosporine and rapamycin) have been explored. Losartan and decorin are potential antifibrotic agents that mitigate TGF-β-induced fibrosis. Ocular nanosystems are innovative drug-delivery platforms that facilitate the targeted release of therapeutic agents. Gene therapies, such as small interfering RNA and antisense oligonucleotides, are promising approaches for selectively inhibiting angiogenesis-related gene expression. Aganirsen is efficacious in reducing the corneal neovascularization area without significant adverse effects. These multifaceted approaches underscore the corneal neovascularization management complexity and highlight ideas for enhancing therapeutic outcomes. Furthermore, the importance of combination therapies and the need for further research to develop specific inhibitors while considering their therapeutic efficacy and potential adverse effects are discussed.

## 1. Introduction

Corneal diseases are one of the most common causes of blindness worldwide, along with age-related macular degeneration (AMD), diabetic retinopathy, and glaucoma. Neovascularization often develops during various corneal diseases and is estimated to occur in 1.4 million people annually, with vision loss in about 12% [1].

A normal cornea is characterized by transparency and a normal refractive surface. The cornea typically lacks blood vessels, and neovascularization can cause impaired vision and other complications. Corneal neovascularization is a disease process caused by corneal hypoxia associated with an imbalance between angiogenic and antiangiogenic factors. Corneal neovascularization is characterized by new blood vessels growing in from the corneal stroma and developing from pre-existing pericorneal vascular structures due to vascular endothelial cell proliferation and migration into the corneal stroma [1,2]. Pathological blood vessels are immature and lack structural integrity. Corneal neovascularization evaluation should focus on the source of vascularization (conjunctival, stromal, iridal), the vessel penetration depth, branching, and leakage that causes lipid exudates and corneal edema. Clinically, afferent vessels are narrower, straighter, in the deeper layers of the cornea, and bright red. Drainage (efferent) vessels have a large caliber and are dark red. Untreated corneal neovascularization is linked to lipid keratopathy due to protein and lipid deposition in the stroma, chronic inflammation, corneal ulceration, corneal edema, and scar formation. Table 1 lists some of the most important factors predisposing a patient to ocular neovascularization [3,4,5].

**Figure 1:**
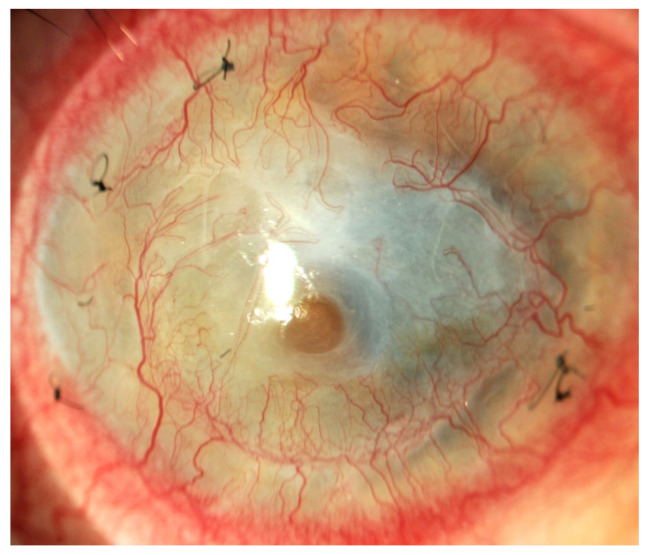
Corneal neovascularization in an autoimmune disease.

**Figure 2:**
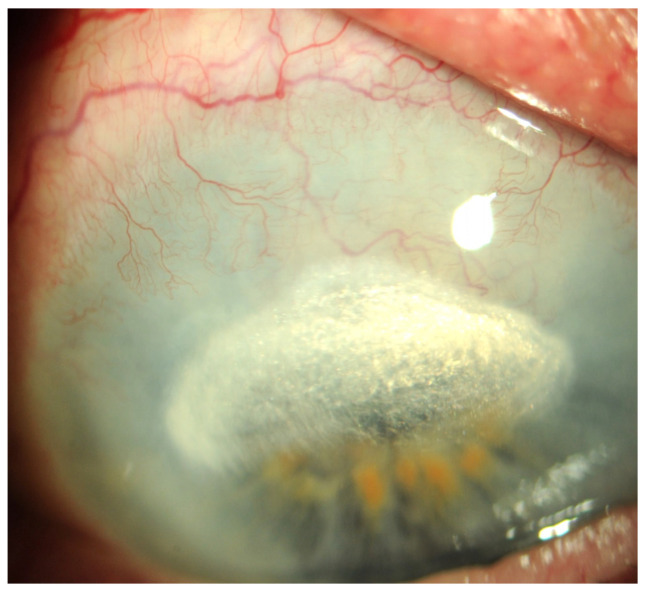
Corneal neovascularization in crystalline keratopathy.

**Figure 3:**
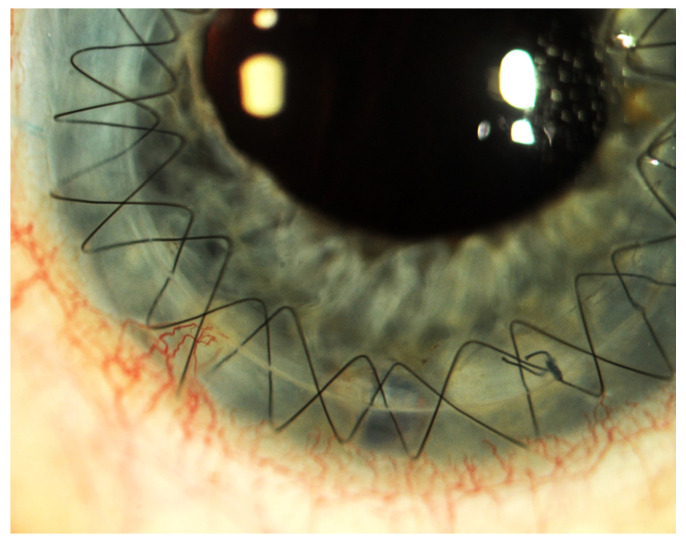
Initial corneal neovascularization after penetrating keratoplasty.

**Figure 4:**
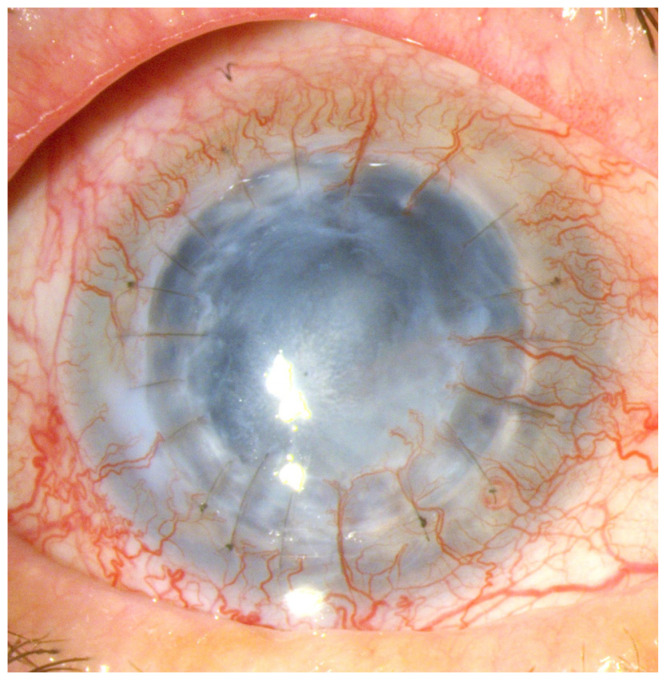
Neovascularization after penetrating keratoplasty.

**Figure 5:**
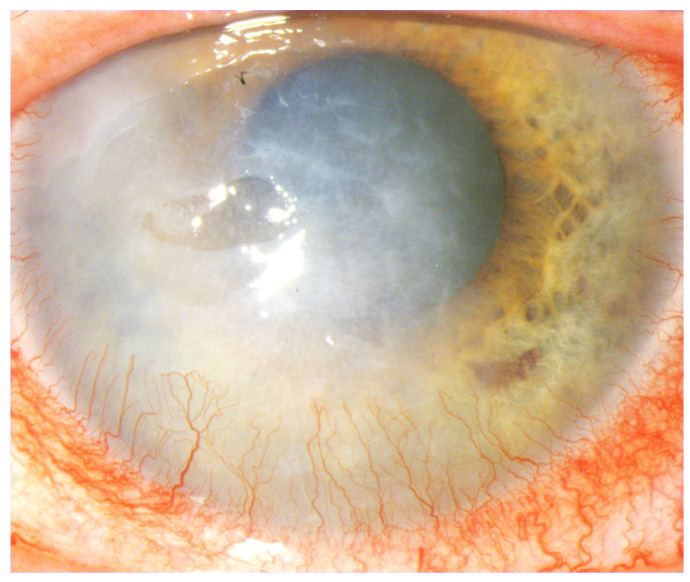
Post-inflammatory corneal neovascularization.

These conditions cause the loss of corneal stem cells, resulting in corneal opacity and significant deterioration of visual acuity up to and including blindness [1,2,6] (Figure 1, Figure 2, Figure 3, Figure 4 and Figure 5).

The pathological factors in corneal neovascularization often involve vascular and inflammatory processes affecting the eye. While these conditions can be distinct, they might share common etiological factors and mutually contribute to progression. The pathophysiological causes of these changes are inflammatory processes and elevated vascular endothelial growth factor (VEGF) levels. Inflammatory responses in the eye can contribute to the growth of new blood vessels. Inflammatory mediators might stimulate angiogenesis in the cornea. VEGF is a key factor in angiogenesis that tends to be elevated in corneal neovascularization. Angiogenesis can cause corneal opacity and edema, which can affect vision and lead to further complications. Treatment approaches include, among others, VEGF inhibitors (anti-VEGF), anti-inflammatory drugs and other therapies, such as surgical procedures [7,8].

The balance between the proangiogenic growth factor (VEGF) and profibrotic connective tissue growth factor (CTGF) influences the angiogenesis and fibrosis equilibrium, referred to as the angio-fibrotic switch. Disrupting this balance after anti-VEGF therapy in favor of CTGF might trigger fibrosis progression. Fibrosis processes in different organs share a common molecular basis. After tissue damage, epithelial cells release mediators that recruit and activate inflammatory cells, endothelial cells, and fibroblasts. Additionally, the cells undergo an endothelial–mesenchymal transition (EMT), a cellular transdifferentiation that converts epithelial cells into myofibroblasts. Neovascularization initiation recruits an increased number of inflammatory cells and fibroblasts, resulting in an additional pool of myofibroblasts. Myofibroblasts produce extracellular matrix (ECM), proliferate, and migrate through the basal layers to cover and regenerate damaged tissue [9].

Neoangiogenesis in retinal diseases exemplifies an imbalance between VEGF and CTGF. Proangiogenic factors often interact to form an intricate signal network that controls angiogenesis. Depending on the physiological or pathological context, different factors might play critical roles in an angiogenesis process.

## 2. Proangiogenic Factors

Angiogenic factors include VEGF, matrix metalloproteinases (MMPs), basic fibroblast growth factors (bFGFs), platelet-derived growth factors (PDGFs), and interleukin-1 (IL-1) [10,11]. VEGF-C and VEGF-D stimulate lymphangiogenesis (lymphatic vessel growth) by interacting with the VEGFR-3 receptor [12,13]. Selected proangiogenic factors are presented and briefly discussed in Table 2.

### 2.1. VEGF

VEGF has numerous biological functions: it participates in blood vessel network formation, exhibits activity against micro- and macrovascular endothelial cells, stimulates endothelial cell proliferation and migration, increases capillary permeability, has neurotrophic activity, participates in tissue inflammatory process development, and regulates gene expression under hypoxia.

Chronic hypoxia induces cellular production of hypoxia-inducible factor (HIF), a transcription factor that stimulates VEGF production and enhances its release. The other biologically active substances that stimulate VEGF synthesis include interleukins (IL-1, IL-5, IL-9, IL-13), tumor necrosis factor alpha (TNF-α), FGF-β, PDGF, transforming growth factor beta (TGF-β), nitric oxide (NO), and reactive oxygen species.

The VEGF family is functionally diverse and consists of VEGF-A, VEGF-B, VEGF-C, VEGF-D, VEGF-E, and the placental growth factor (PlGF). VEGF-A is involved in angiogenesis, vasodilation, and NO release and enhances macrophage and granulocyte chemotaxis. VEGF-B participates in neovascularization during embryonic development and in tumor growth progression. VEGF-C enhances vascular permeability and is involved in lymphangiogenesis. VEGF-D affects blood and lymphatic vessel remodeling and significantly influences disease processes [14].

Macrophages, T lymphocytes, astrocytes, pericytes, retinal pigment epithelial cells, and smooth muscle cells produce VEGF in response to hypoxia and inflammatory processes. In the human body, VEGF exists in at least six isoforms: VEGF121, VEGF145, VEGF165, VEGF183, VEGF189, and VEGF206. All VEGF family proteins stimulate a cellular response by binding to tyrosine kinase receptors (VEGFRs) on the cell surface, resulting in their dimerization and activation by transphosphorylation. VEGF-A binds to VEGFR-1 (FLT-1) and VEGFR-2 (KDR/FLT-1) receptors. VEGFR-2 mediates almost all known cellular responses to VEGF. VEGFR-1 function has not been defined, but it is thought to modulate the VEGFR-2 signaling action. VEGFR-3 does not bind to VEGF-A. Its ligands are VEGFR-C and VEGFR-D, and it mediates lymphangiogenesis [13,15].

VEGF-A is the most significant VEGF factor in corneal neovascularization and is secreted by a wide variety of heterogeneous cells, including macrophages, T lymphocytes, fibroblasts, pericytes, astrocytes, retinal pigment epithelial cells, corneal epithelial cells, corneal endothelial cells, and corneal keratocytes. VEGF-A binds to tyrosine kinase receptors, i.e., VEGFR-1 and VEGFR-2. VEGF-C and VEGF-D can be secreted by macrophages in the corneal stroma during inflammatory or traumatic processes [4,16].

### 2.2. Extracellular Matrix Metalloproteinases (MMPs)

Extracellular matrix metalloproteinases (MMPs) are enzymes vital in the degradation and remodeling of extracellular matrix (ECM) components in physiological and pathological processes, including tissue development, wound healing, and inflammation. The regulation of tissue inhibitors of metalloproteinases (TIMPs) and the balance between TIMPs and MMPs is crucial for maintaining tissue homeostasis. Disrupting this balance can lead to excessive ECM degradation and neovascularization. MMPs are involved in the corneal neovascularization process. Their excessive production can lead to ECM barrier degradation and destruction, allowing endothelial cell migration and creating a pathway for new blood vessel growth in the corneal stroma. Growth factor and cytokine release regulate cell migration and proliferation. MMP involvement in corneal neovascularization suggests that targeting these enzymes may be a potential strategy for treating conditions in which corneal blood vessel growth becomes an issue, e.g., when MMP14 interacts with VEGFR1 to stimulate VEGF-A-induced angiogenesis. However, the exact roles of specific MMPs in corneal neovascularization might vary with the underlying condition. Therefore, further studies are needed to fully understand their roles and potential therapeutic interventions [11].

### 2.3. bFGF

The FGF family is a group of pleiotropic factors that affect vascular endothelial cells. Their ligands are heparan sulfate proteoglycans (HSPGs) and the FGF receptor (FGFR1-4) family, which exhibits tyrosine kinase activity like the VEGFRs. FGF-1 expression occurs in an intact corneal epithelium, while FGF-2 is markedly overexpressed following corneal injury. Receptor autophosphorylation occurs when FGF-1, FGF-2, and FGF-4 bind to their FGFRs, activating the Shc adapter protein, proximal adapter protein FRS2, and adapter molecule crk. Hence, endothelial cell proliferation leading to angiogenesis becomes possible by mitogen-activated protein kinase (MAPK) cascade activation and by blocking protein kinase C (PKC) activation [17]. FGFR-1 promotes the ECM degradation that precedes angiogenesis [7,18]. FGFR-2 and FGFR-4 greatly enhance endothelial cell production of urokinase-type plasminogen activator (uPa), facilitating new vasculature formation. FGF-2, FGF-8, and FGF-10 isoforms cause chemotaxis, endothelial cell migration, and proliferation [19]. bFGF induces corneal angiogenesis by regulating VEGF-A, VEGF-C, and VEGF-D expression [20,21].

### 2.4. IL-1

IL-1 is a proinflammatory cytokine produced by various cells, including fibroblasts, macrophages, and neutrophils. IL-1 induces the expression of adhesion molecules, chemokines, and growth factors, all leading to neovascularization. IL-1 is significant in immune responses and inflammatory processes. IL-1α and IL-1β might contribute to corneal neovascularization through several mechanisms. IL-1 stimulates the production of proangiogenic factors, such as VEGF and bFGF, which promote new blood vessel growth. Furthermore, IL-1 stimulates inflammatory cell migration and adhesion to the injury or inflammation site.

IL-1 increases the production of metalloproteinases, which degrade the ECM and create space for new blood vessel growth. IL-1 activates vascular endothelial cells, leading to their migration and increased vascular permeability, facilitating the corneal neovascularization process. IL-1 might also be involved in lymphangiogenesis, contributing to the generalized neovascularization process. Given these mechanisms, IL-1 has been identified as a potential target for interventions to prevent or treat corneal neovascularization [22].

### 2.5. PDGF

PDGF is associated with corneal neovascularization and is involved in tissue remodeling, including cell growth and division, and angiogenesis. PDGF is secreted by various cells, including platelets, endothelial cells, and macrophages. PDGF-A, PDGF-B and their complementary receptors (PDGFRA and PDGFRB, respectively) are associated with corneal neovascularization [23].

## 3. Antiangiogenic Factors

The antiangiogenic agents include endostatin and endostatin analogs (neostatin, canstatin, tumstatin), plasminogen activator inhibitor-1 (PAI-1), and serine protease inhibitors [retinal pigment epithelium-derived factor (PEDF), angiostatin, thrombospondin 1, 2 (TSP-1, TSP-2, respectively), and interferon (IFN)-α]. Selected antiangiogenic factors are briefly discussed in Table 3.

### 3.1. Endostatin

Endostatin belongs to the collagen family and is most frequently localized in perivascular tissue. Endostatin is a fragment of the NC1 domain of type XVIII collagen. Collagen XVIII is expressed in developing ocular tissue and basement membranes and exhibits potent antiangiogenic properties. First, NC1 is digested by metalloproteinases (MMP3, MMP7, MMP9, MMP12, MMP13, MMP20), then cathepsin L acts on the resulting fragments, forming mature endostatin. Endostatin binds to tropomyosin, integrins, soluble VEGFR (sVEGFR), glypicans, and metalloproteinases to inhibit endothelial cell migration and proliferation. Endostatin blocks VEGF-dependent KDR/flk1 phosphorylation and ERK, p38, MAPK, and p125 activation, which are crucial for endothelial cell proliferation and migration. Additionally, endostatin activates caspase-3, an intracellular protease that enhances endothelial cell apoptosis, counteracting angiogenesis [20].

### 3.2. PAI-1

Under certain conditions, increased PAI-1 levels can reduce the expression of VEGF, a key driver of angiogenesis, including in the cornea. Therefore, higher PAI-1 levels might limit new blood vessel growth. The relationship between PAI-1 and corneal neovascularization is complex and can vary with the specific context, such as the underlying cause of neovascularization, the overall balance of pro- and antiangiogenic factors, and the individual’s health status [24].

### 3.3. PEDF

PEDF is also known as serpin F1 (SERPINF1) and is a multifunctional protein with antiangiogenic, antitumorigenic, and neurotrophic functions. PEDF binds to the receptor PEDF-R on the cell surface. PEDF-R acts against phospholipase A2, whose activation leads to the release of fatty acids from glycerolipids. PEDF increases gamma-secretase activity, cleaving the VEGFR-1 transmembrane domain. This action interferes with VEGF signaling, inhibiting angiogenesis [25]. Administering PEDF to the cornea inhibits neovascularization, a much more pronounced effect than that of other antiangiogenic agents such as angiostatin or thrombospondin. The laminin receptor is also a PEDF target, where the interaction occurs between PEDF residues 24–57, a region that regulates the antiangiogenic function [26].

### 3.4. Angiostatin

Angiostatin is an endogenous antiangiogenic factor also produced in the cornea. Angiostatin is a fragment of plasminogen, a precursor molecule involved in fibrinolysis. Angiostatin attaches to endothelial surface proteins and inhibits the neovascularization process. Due to its ability to inhibit new blood vessel growth, angiostatin has been investigated as a potential therapeutic agent for corneal neovascularization. The main challenge is the difficulty of delivering angiostatin to the cornea stably and efficaciously. Additionally, the potential side effects and long-term safety of angiostatin must be carefully evaluated [27].

### 3.5. Thrombospondin

In 2009, the Wilmer Eye Institute examined the role of thrombospondin in inhibiting corneal neovascularization. The effect of wispostatin-1 (WISP-1), a peptide derived from TSP-1, on corneal neovascularization caused by topical administration of bFGF and laser-induced choroidal neovascularization was investigated. WISP-1 demonstrated potent inhibitory effects on endothelial cell migration and proliferation in vitro. In vivo, the test peptide completely inhibited bFGF-induced neovascularization and largely inhibited laser-induced choroidal neovascularization. These encouraging results revealed the potential of using WISP-1 to treat corneal diseases [20,28].

### 3.6. IFN-α

IFN-α is a cytokine crucial in regulating immune processes. IFN-α has anti-inflammatory and antiangiogenic properties, potentially inhibiting new blood vessel growth and reducing corneal inflammation. Using IFN-α in corneal neovascularization is not a well-established or widely accepted treatment option. Some studies and reports demonstrated that topical or subconjunctival administration of IFN-α might reduce corneal neovascularization. However, the evidence is limited, and further studies are needed to determine the safety, efficacy, and optimal dosing protocols [29].

## 4. Treatment of Corneal Neovascularization

Treatment of corneal neovascularization is either topical (steroid and non-steroidal anti-inflammatory drugs [NSAIDs], cyclosporine A, anti-VEGF drops) or surgical (laser photocoagulation, diathermy/fine needle cauterization, superficial keratectomy, subconjunctival injections of anti-VEGF drugs, amniotic membrane transplantation, corneal transplantation). However, corneal neovascularization treatment is not always effective and can produce side effects.

### 4.1. Steroids

Corneal neovascularization may lead to decreased visual acuity as a result of edema, persistent inflammation, intrastromal deposition of proteins and lipids, and the formation of corneal fibrosis.

Fibrosis is characterized by the excessive accumulation of ECM proteins, primarily collagen, leading to tissue scarring and impaired organ function. Steroids, specifically glucocorticoids, have been used to treat fibrotic disorders due to their anti-inflammatory and immunosuppressive properties. Some of the indirect mechanisms by which steroids influence the fibrotic process are described below.

Anti-inflammatory effects: Inflammation is a key contributor to fibrosis development and progression. Steroids suppress the inflammatory response by inhibiting the activity of immune cells, such as macrophages and lymphocytes, and reduce the production of inflammatory mediators, including cytokines and chemokines. Therefore, steroids prevent or limit the fibrotic process by dampening inflammation.

Inhibiting fibroblast activation: Fibroblasts produce collagen and other ECM components. Steroids interfere with fibroblast activation and proliferation, preventing the synthesis of excess collagen. This fibroblast activity modulation contributes to the antifibrotic effects of steroids.

Modulating cytokines and growth factors: Steroids influence the production and activity of cytokines and growth factors regulating fibrosis, e.g., TGF-β, indirectly regulating the fibrotic response.

Immunosuppression: Fibrosis can be associated with an autoimmune or other inflammatory conditions. Steroids are immunosuppressive agents that control this immune response and reduce tissue damage. This immunosuppressive effect is particularly relevant in fibrotic disorders with an autoimmune component, such as systemic sclerosis [17,18,19,30].

Corneal neovascularization is typically accompanied by an inflammatory process. Therefore, steroidal anti-inflammatory drugs, especially corticosteroids with anti-inflammatory and immunosuppressive properties, are important. Applied in drops into the conjunctival sac, corticosteroids reduce the local immune response and inhibit the growth of new blood vessels. Subconjunctival corticosteroid injections can be considered in some cases as they provide higher drug concentrations directly to the affected area, potentially leading to better outcomes with fewer systemic side effects. However, long-term topical steroid use can cause side effects, including glaucoma, cataract formation, and bacterial and fungal superinfections. Furthermore, steroids have limited antiangiogenic effects, as they do not reduce mature corneal neovascularization [31].

A potent steroid drug is a 0.1% dexamethasone suspension administered as 1–2 drops into the conjunctival sac. Dexamethasone is a synthetic glucocorticosteroid with long-lasting anti-inflammatory, anti-edema, anti-allergic, and immunosuppressive effects. Dexamethasone affects all inflammatory process phases, inhibits leukocyte migration, blocks IgE-dependent histamine and leukotriene secretion, inhibits cytokine synthesis and release, inhibits phospholipase A2 activity, prevents arachidonic acid release and inflammatory mediator synthesis (leukotrienes and prostaglandins), inhibits phagocytic cell migration in response to an inflammatory stimulus, inhibits kinin release and antibody production, and reduces capillary permeability and edema [32,33]. Dexamethasone (0.1%) is administered into the conjunctival sac and is absorbed into the aqueous fluid, cornea, iris, choroid, ciliary body, and retina. Absorption of the active ingredient from the conjunctival sac into general circulation is minimal such that its pharmacokinetics and systemic effects are clinically insignificant.

Steroid drugs also have adverse effects and can contribute to the development of infections, steroid-induced glaucoma, and posterior chamber cataracts. They can also promote herpes simplex recurrence [34].

Topically administered steroid drugs also include 0.5% loteprednol etabonate. Loteprednol etabonate is synthesized by structural modification of prednisolone derivative compounds and belongs to the topical corticosteroids, which are transformed into inactive carboxylic acid derivatives. Loteprednol etabonate has a potent anti-inflammatory effect but has little effect on intraocular pressure and steroid-induced glaucoma development. The intraocular pressure increases after a much longer administration period compared to prednisolone acetate. In clinical trials, only 1.7% of patients experienced an increased intraocular pressure (≥10 mm Hg). A few patients demonstrated significant intraocular pressure elevation, but this quickly returned to normal after the drug was discontinued [35].

A 0.1% aqueous solution of betamethasone acetate is used topically. A higher concentration, i.e., 0.2%, has a potency similar to dexamethasone, a better safety profile, and requires less frequent administration. Furthermore, its effect persists for several hours. Fluorometholone (0.1% suspension) is also administered into the conjunctival sac, as is prednisolone sodium phosphate (0.5% and 1.0% drops). Other topical corticosteroids include 0.05% difluprednate, a 1.0% loteprednol etabonate suspension, and a 0.38% loteprednol etabonate ophthalmic gel [36]. The latter reduces the number of inflammatory cells in the anterior chamber and significantly alleviates ocular pain (74.4%), with only a minimal intraocular pressure elevation [37].

Oral systemic corticosteroids can be used where corneal neovascularization is associated with systemic inflammation, autoimmune diseases or, in graft disease, corneal transplant rejection. However, systemic steroid use is associated with several potential side effects. Therefore, the benefit–risk balance should be considered with care. Prednisone and methylprednisolone are the most commonly used oral steroids for treating ophthalmic conditions.

Prednisone is a synthetic glucocorticosteroid that is a derivative of cortisol, an inactive compound metabolized in the liver to active prednisolone with potent anti-inflammatory effects. Prednisone reduces the accumulation of leukocytes and their adhesion to the capillary vessel endothelium; inhibits phagocytosis and lysosome breakdown; reduces lymphocyte, eosinophil, and monocyte numbers; and blocks IgE-dependent histamine and leukotriene secretion. Prednisone also inhibits the synthesis and release of the following cytokines: IFN-γ, IL-1, IL-2, IL-3, IL-6, TNF-α, and granulocyte-macrophage colony-stimulating factor (GM-CSF). By inhibiting phospholipase A2 activity via lipocortin, prednisone prevents arachidonic acid release and inflammatory mediator (leukotriene and prostaglandin) synthesis. Prednisone decreases tissue edema by reducing capillary permeability.

Prednisone also exhibits immunosuppressive effects. The mechanisms of immunosuppressive action are not entirely understood. Nevertheless, prednisolone prevents or inhibits cellular immune responses and specific mechanisms related to the immune response. Prednisone reduces T lymphocyte, monocyte, and acidophilic granulocyte numbers and reduces immunoglobulin binding to receptors on the cell surface. Prednisone inhibits interleukin synthesis or release by decreasing T-lymphocyte blastogenesis and reducing the severity of the early immune response. Prednisone also impedes immune complex penetration across basement membranes and reduces complement component and immunoglobulin concentrations. The complications of prednisone use include highly refractive, multicolored corneal deposits mainly in the sub-epithelium and anterior corneal stroma [38].

Methylprednisolone aceponate produces a greater anti-inflammatory effect, causes less sodium and water retention than prednisone, and is at least four times more potent than hydrocortisone. Methylprednisolone aceponate mainly acts intracellularly at the DNA level, which delays its effects. Methylprednisolone aceponate reduces the accumulation of leukocytes and their adhesion to the endothelium; inhibits phagocytosis and lysosome breakdown; reduces lymphocyte, eosinophil, and monocyte numbers; blocks IgE-dependent histamine and leukotriene secretion; inhibits the synthesis and release of cytokines such as IFN-γ, IL-1, IL-2, IL-3, IL-6, and TNF-α; inhibits phospholipase A2 activity; and prevents arachidonic acid release and consequent inflammatory mediator synthesis. Methylprednisolone aceponate also inhibits capillary permeability and reduces edema. Long-term use with low-maintenance doses is often necessary in treating chronic diseases. Such a regimen aims to ensure a favorable profile of glucocorticosteroids and minimize adverse effects, such as hypothalamic–pituitary–adrenal axis inhibition or Cushing’s syndrome.

Long-term corticosteroid use can cause posterior subcapsular and nuclear cataracts, especially in children, an increased intraocular pressure, and glaucoma. In most studies reviewed, ocular hypertension developed in children within 1 month of starting steroid treatment [39]. With prolonged steroid therapy, the prevalence of secondary fungal and viral eye infections increases, and central serous chorioretinopathy (CSR) might develop [19,40].

### 4.2. NSAIDs

NSAIDs have been studied for their potential role in managing corneal neovascularization. NSAIDs primarily work by inhibiting cyclooxygenase (COX), which is involved in prostaglandin synthesis. NSAIDs might have antiangiogenic effects, hindering new blood vessel formation. However, the evidence for NSAID effectiveness in managing corneal neovascularization is less robust than that for other treatments. In this context, NSAID use might vary with the underlying cause of neovascularization. For this reason, NSAIDs are used mainly to stop the inflammatory process that may lead to corneal neovascularization. NSAIDs should be considered as an adjunct treatment for corneal neovascularization, rather than as a principal agent.

Some studies indicated that NSAIDs inhibit the angiogenic process, potentially slowing or preventing new blood vessel growth in the cornea by downregulating VEGF production [41]. COX-2 is implicated in promoting angiogenesis under certain conditions. NSAIDs inhibit the activity of COXs, which participate in prostaglandin synthesis and promote angiogenesis. Inhibiting COX-2 through NSAIDs might therefore contribute to antiangiogenic effects [42] by inhibiting prostaglandin production, indirectly inhibiting angiogenesis [43].

NSAIDs can be administered topically as eye drops, considered safe and commonly used for managing ocular inflammation. However, topical NSAIDs might have limited efficacy for corneal neovascularization. Therefore, NSAIDs may be combined with other treatments, such as corticosteroids or anti-VEGF agents [44]. Nevertheless, NSAIDs should be used with caution as they can cause corneal ulceration and melting [45] and neurotrophic keratitis [46].

Bromfenac sodium (0.09%) ophthalmic solution is a clinically useful topical NSAID. Bromfenac exerts anti-inflammatory effects by inhibiting COX-2 and blocking prostaglandin synthesis. Bromfenac inhibited corneal neovascularization formation and reduced VEGF and COX-2 expression in corneal tissue after alkali burns [47]. Additionally, bromfenac significantly decreased VEGF and monocyte chemoattractant protein-1 levels [48].

Nepafenac (0.1% and 0.3%) is another topical NSAID that is an amphenac prodrug. Nepafenac topically administered into the eye penetrates the cornea and rapidly bioactivates to amfenac, a potent NSAID. The nepafenac anti-inflammatory effect consists of inhibiting COX-2 and blocking prostaglandin synthesis. Chronic nepafenac use can lead to corneal epithelial damage, thinning, ulceration, or corneal perforation [49].

The most widely used NSAID is diclofenac sodium, an aminophenylacetic acid derivative with potent anti-inflammatory, analgesic, and antipyretic effects. The drug action is mainly based on the inhibition of COXs involved in synthesizing prostaglandins, which mediate inflammation. Diclofenac has a greater inhibitory effect on COX-1, a constitutive enzyme engaged in prostaglandin synthesis with physiological functions, than on inducible COX-2, which is responsible for proinflammatory prostaglandin synthesis at the inflammation site. Additionally, it reversibly inhibits platelet aggregation stimulated by adenosine diphosphate (ADP) and collagen. The most common adverse effect of diclofenac sodium is a transient mild- to moderate-intensity burning sensation in the eyes. Corneal epithelium damage and punctate keratitis development may occur during long-term use of diclofenac eye drops. Due to its synergistic anti-VEGF and anti-inflammatory effects, a hydrogel combining diclofenac sodium with bevacizumab has better antiangiogenic effects than a drug containing bevacizumab alone [50]. Therefore, NSAIDs may be an effective adjunct to the treatment of corneal neovascularization.

### 4.3. VEGF Inhibitors

**Figure 6:**
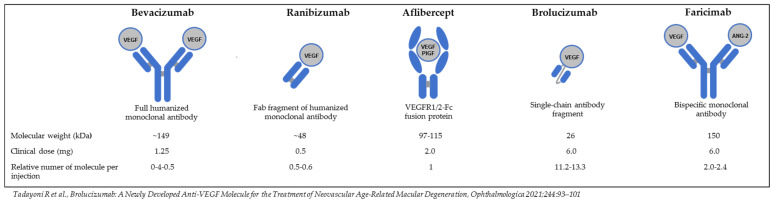
Molecular characteristics and clinical dosing of anti-VEGF agents [52].

VEGF inhibitors are vital in the angiogenesis process. Anti-VEGF preparations used as drops or subconjunctival injections, such as bevacizumab, aflibercept, and ranibizumab, bind to VEGF and prevent interaction with its receptors, reducing the angiogenic response. Newly formed vessels exhibit a good response to treatment with anti-VEGF agents in contrast to mature vessels in chronic neovascularization, on which these drugs are less effective due to abundant pericyte recruitment to the angiogenesis sites. Therefore, anti-VEGF therapy targets new blood vessels, is a symptomatic treatment, and requires repetition to maintain efficacy over time [51]. Currently, there are several commonly used anti-VEGF drugs. Figure 6 graphically shows the structure of anti-VEGF drugs, starting with the oldest bevacizumab, then ranibizumab, aflibercept, brolucizumab, and the newest faricimab, with which we have no experience yet in the treatment of corneal neovascularization [52].

Ranibizumab is a fragment of a recombinant humanized monoclonal antibody produced in *Escherichia coli* cells using recombinant DNA technology. Ranibizumab has a molecular weight of 48 kDa and high affinity for human VEGF-A. Ranibizumab binds to and therefore blocks all VEGF-A isoforms (VEGF110, VEGF112, VEGF165), preventing VEGF-A from binding to its receptors VEGFR-1 and VEGFR2. This result is important as VEGF-A binding to receptors leads to endothelial cell proliferation and new vessel formation. As ranibizumab is an antigen-binding Fab fragment without an Fc domain, its size is approximately one-third of that of bevacizumab. Therefore, ranibizumab might have better penetration into the cornea than bevacizumab [53].

Ranibizumab exhibits antiangiogenic properties by simultaneously suppressing blood and lymphatic vessel growth, highlighting its potential in treating corneal neovascularization [54]. In drop form, topical 1% ranibizumab reduces the stable neovascularization area and decreases the vessel diameter while not altering vessel length [55]. Additionally, subconjunctival ranibizumab significantly decreases VEGF levels in aqueous fluid, reducing the neovascularization area at the iridocorneal angle and iris. Therefore, ranibizumab might also be used to treat NVG [56].

Aflibercept, referred to in the literature as “VEGF trap”, is a recombinant fusion protein consisting of the VEGFR-1 and VEGFR-2 extracellular components fused to the human IgG1 Fc fragment. Aflibercept is produced by recombinant DNA technology and is a glycoprotein dimer with a molecular weight of 115 kDa. Aflibercept binds to circulating VEGF, thereby “trapping” it and inhibiting VEGF-A, VEGF-B and PlGF activity, suppressing new blood vessel growth. Aflibercept inhibits bFGF-induced corneal neovascularization and can be used in eyes previously treated with bevacizumab or ranibizumab [57]. A comparative analysis of the cytotoxic effects of bevacizumab, ranibizumab, and aflibercept demonstrated that ranibizumab and aflibercept cause less corneal epithelial damage in patients with pre-existing corneal epithelial lesions [58,59]. Aflibercept can be administered as conjunctival drops or subconjunctivally. Subconjunctival injection can be repeated after 1–2 months, depending on the clinical effect [60].

Brolucizumab is a humanized monoclonal single-chain antibody fragment (scFv) produced by recombinant DNA synthesis in the *E*. *coli* cytoplasm. It acts as a VEGF inhibitor. The US Food and Drug Administration approved the drug for treating wet AMD in 2019. Reports on using brolucizumab eye drops or subconjunctival injections for treating corneal neovascularization have not been published.

Bevacizumab is a recombinant humanized monoclonal antibody produced using DNA technology. Topical, subconjunctival, and intraocular bevacizumab reduces corneal neovascularization and improves corneal transparency. Furthermore, bevacizumab reduces the vascular diameter and neovascularization area by 24% and 61%, respectively [61]. Maximum effects are observed with early topical administration. Subconjunctival bevacizumab injections were also effective in treating corneal neovascularization [62]. A comparison between subconjunctival administration and topical application of bevacizumab demonstrated both methods effectively inhibit corneal angiogenesis and reduce inflammation. However, there was concern that topical bevacizumab might weaken corneal epithelial adhesion to the basement membrane, delaying wound healing and causing corneal stroma thinning. These adverse effects increase with higher doses (>1.0%) and longer treatment durations (>1 month) [63,64]. Topical 1.25% bevacizumab causes epitheliopathy in the second month of treatment, but 0.5% bevacizumab results in a low prevalence of corneal epithelial defects [65,66].

Anti-VEGF drugs are used to stop the rejection of the transplanted corneal flap. The high success rate of penetrating keratoplasty is overshadowed by the high rejection rate of >50% in the case of corneal transplantation on a vascularized and inflamed cornea; these are “high risk” procedures because of the risk of immune-related rejection despite aggressive immunosuppressive therapy. Corneal transplant failure is most often caused by immune-related rejection of the transplanted tissue. The immunopathological mechanisms disrupting corneal immune privilege have not been fully defined, but evidence clearly suggests that increased allosensitization and subsequent graft rejection are associated with the presence of corneal neovascularization. Therefore, strategies to inhibit corneal neovascularization have the potential to inhibit the alloimmune response and prevent corneal graft rejection [67]. Several preclinical studies have highlighted the effectiveness of bevacizumab in inhibiting corneal neovascularization by neutralizing VEGF-A [68,69].

Dohlman et al. assessed the clinical effectiveness of bevacizumab in inhibiting corneal neovascularization after high-risk corneal transplantation over a 52-week period. Topical and subconjunctival bevacizumab treatment significantly reduced the area of corneal vascularization and areas of neovascularization invasion into the transplanted corneal flap. The primary efficacy outcome measures in this study were areas of vascularity and invasion in the graft tissue and host beds.

In patients who received bevacizumab, the vascular area in the control group was significantly reduced at week 52 after surgery. The vascular area was comparable in both groups on postoperative day 1, while at 1 week, bevacizumab-treated patients had a moderately smaller vascular area compared with vehicle controls. However, the vascular area in the bevacizumab group was significantly smaller at subsequent time points (weeks 4, 8, 16, 26, 39, and 52). The authors also observed a moderate reduction in vascular area in patients from the control group, attributing this change to the intensive topical corticosteroid treatment that all patients received due to the high-risk categorization of corneal transplantation. Therefore, the significant reduction in the vascular area observed in the bevacizumab group was detectable despite the intensive steroid regimen.

Interestingly, the authors observed similar reductions in the vascular area in bevacizumab-treated patients after 52 weeks, regardless of whether patients underwent primary or repeat corneal transplantation. However, in the vehicle group, the vascular area was larger in patients undergoing repeated corneal transplantation compared to those undergoing the procedure for the first time [67].

Bhatti et al. assessed the antiangiogenic effect of bevacizumab in high-risk corneal transplants, showing a significant reduction in the area of corneal vascularization after treatment [66,70]. In another study, Krizova et al. reported 38.04% and 22.7% reductions in the vascular area in the peripheral and central regions, respectively. Corneal segments in bevacizumab-treated patients only showed 21.6 and 9.6% reductions in the vascularized area in the central and peripheral corneal segments, respectively, in the control group [51].

Eski et al. investigated the effect of subconjunctival bevacizumab, ranibizumab, and aflibercept on the process of corneal neovascularization in an experimental model. The examined eyes were randomly divided into four groups: a bevacizumab group (0.05 mL/1.25 mg bevacizumab), a ranibizumab group (0.05 mL/0.5 mg ranibizumab), an aflibercept group (0.05 mL/1.25 mg aflibercept), and a control group (0.05 mL of saline solution). The plasma VEGF level was one of the main outcomes measured to assess corneal neovascularization. In the period after subconjunctival administration, the number of main corneal arteries in the injection groups was significantly reduced compared to the control group, but there were no statistically significant differences between the injection groups. The aflibercept group had the lowest area of neovascularization. Immunohistochemical staining revealed significantly lower percentages of VEGF in neovascularized arteries in the drug groups than in the control group. No statistically significant differences were observed between groups in terms of the corneal surface, with an increased number of cells and increased amounts of edema and inflammation. The authors concluded that subconjunctival bevacizumab, ranibizumab, and aflibercept appeared effective in inhibiting corneal neovascularization without causing epitheliopathy compared to the control group. However, no significant results were reported between these three anti-VEGF molecules [71].

Corneal transparency is maintained by preventing the vascularization of the stroma. VEGF secreted by keratocytes promotes not only neovascularization but also the proliferation and metaplasia of epithelial progenitor cells in the central cornea. Inhibition of VEGF through a pathway involving Notch1 and Hif1a proteins maintains a clear corneal stroma and remains a possible treatment option for certain conditions associated with corneal neovascularization [72].

Despite the extensive literature confirming the effectiveness of treating corneal neovascularization with subconjunctival injections or drops of anti-VEGF drugs, the treatment is off label.

### 4.4. Immunosuppressive Drugs

Cyclosporine is an immunosuppressive drug that has been studied for its effects on angiogenesis in various contexts, particularly in transplantation- and immune-related disorders. The relationship between cyclosporine and angiogenesis is complex, and the drug can have proangiogenic and antiangiogenic effects depending on the conditions and cell types involved.

Cyclosporine A is usually used at a concentration of 0.1%. Occasionally, prescription drops are prepared at a higher concentration of 0.5–2% when the corneal disease process is severe or at 0.05–0.1% when there are clinical indications for lower concentrations. Cyclosporine A inhibits the production or release of proinflammatory cytokines, including IL-2 [T-cell growth factor (TCGF)]. Cyclosporine A also specifically and reversibly inhibits lymphocytes in the G0 or G1 phase of the cell cycle. After administration into the conjunctival sac, cyclosporine A is passively absorbed by T lymphocytes, infiltrating the cornea and conjunctiva and inactivating calcineurin phosphatase; inhibiting the dephosphorylation of the transcription factor NF-AT and preventing NF-AT translocation into the nucleus; and blocking the release of proinflammatory cytokines such as IL-2. Hence, cyclosporine A is an immunosuppressive drug that inhibits the immune system response, particularly T lymphocyte activation [73].

The anti-inflammatory and immunosuppressive properties of cyclosporine might control the angiogenic process in corneal neovascularization. Cyclosporine-containing eye drops allow for direct application of the drug to the affected area to inhibit new blood vessel growth and reduce cornea inflammation. Topical 0.05% cyclosporine causes corneal stromal neovascularization regression and reduces the corneal graft rejection risk in selected cases. Contrastingly, topical treatment with 0.1% cyclosporine is more effective in inhibiting newly formed corneal neovascularization than bevacizumab eye drops applied to the conjunctival sac [73,74]. Cyclosporine is not a first-line treatment for corneal neovascularization. Depending on the cause of the neovascularization and the patient’s overall condition, other therapeutic options such as corticosteroids, anti-VEGF preparations, and surgical interventions should be considered [74,75].

Some studies suggested that cyclosporine might have proangiogenic effects during wound healing. Cyclosporine might promote new blood vessel formation in tissue repair [76]. Cyclosporine inhibits the expression of VEGF, a key factor in promoting angiogenesis. This antiangiogenic effect might contribute to the cyclosporine ability to reduce graft rejection in transplant recipients [77]. Furthermore, the influence of cyclosporine on angiogenesis might be dose-dependent. Different drug concentrations might lead to varied effects on blood vessel formation. Notably, the concentration, exposure duration, and specific cellular and tissue contexts can influence the effects of cyclosporine on angiogenesis. The balance between proangiogenic and antiangiogenic effects might vary, and the clinical implications of these effects should be considered in the specific medical conditions for which cyclosporine is prescribed [78].

Rapamycin (sirolimus) is a macrolide antibiotic immunosuppressant that effectively reduces angiogenesis probably by inhibiting proinflammatory cytokines. Accordingly, rapamycin could be used to regulate angiogenesis in treating corneal diseases manifested by neovascularization. Rapamycin is effective in treating herpes simplex virus 1 (HSV-1)-induced interstitial keratitis [79]. Rapamycin effectively downregulates mRNA expression levels of TNF-α and IL-1β. It also inhibits neutrophil and macrophage infiltration and suppresses inflammation-related angiogenesis mediated by MMP2. Furthermore, rapamycin inhibits inflammation induced by corneal alkali burns by regulating angiogenesis through HIF-1α–VEGF and the serum cytokines TNF-α, IL-6, IFN-γ, and GM-CSF [80].

Rapamycin is an immunosuppressive drug with additional anti-proliferative properties. It is a specific inhibitor of mammalian target of rapamycin (mTOR) and has been studied for its effects on angiogenesis. The influence of rapamycin on angiogenesis is complex and might depend on the conditions, concentrations, and cell types involved. Rapamycin primarily acts by inhibiting the mTOR pathway, leading to antiangiogenic effects by suppressing the activity of endothelial cells involved in blood vessel formation [81]. Rapamycin also inhibits VEGF production in certain contexts, indicating a potential antiangiogenic influence [82].

Rapamycin has a dual role in angiogenesis, with antiangiogenic effects in some contexts (cancer) and potentially proangiogenic effects in others (wound healing). The dual effects of rapamycin on angiogenesis highlight its context-dependent actions and the importance of considering specific conditions and cell types. The clinical implications of rapamycin’s effects on angiogenesis require careful evaluation and might depend on the medical context, such as organ transplantation, cancer therapy, or wound healing [83].

Tacrolimus is used in conjunctival drops to inhibit corneal graft rejection due to neovascularization. Tacrolimus is safe and effective in improving graft survival in high-risk patients [84,85]. Furthermore, it effectively prevents irreversible corneal graft rejection in high-risk patients [86].

Tacrolimus is an immunosuppressive drug commonly used in transplantation to prevent graft rejection. Various studies investigated the influence of tacrolimus on angiogenesis and suggested that tacrolimus might exhibit proangiogenic and antiangiogenic effects depending on the context and experimental conditions [87]. Its antiangiogenic effect might contribute to its immunosuppressive properties. Some studies suggested that tacrolimus might have proangiogenic effects and contribute to improved wound healing. Tacrolimus enhances new blood vessel formation during tissue repair and also inhibits VEGF production. The influence of tacrolimus on angiogenesis may be dose-dependent, with varying responses at different drug concentrations. Tacrolimus influences endothelial cell function, affecting migration and tube formation, which are crucial for angiogenesis [88]. The concentration, duration of exposure, and the specific experimental models used can influence the effects of tacrolimus on angiogenesis [89].

### 4.5. Other Therapies

Losartan is an angiotensin II receptor blocker (ARB) commonly used to treat several conditions, including hypertension. Losartan has been studied for its potential antifibrotic effects in various tissues, including the cornea. Topical losartan is likely to effectively reduce corneal scarring fibrosis produced by traumatic injury, microbial infections, and some corneal diseases and surgeries. Losartan inhibits the activity of TGF-β, a key cytokine in fibrotic processes. TGF-β signaling is important in corneal fibrosis; therefore, losartan’s inhibition of the pathway might mitigate fibrotic changes. TGF-β stimulates collagen synthesis, particularly collagen types I and III, major ECM components. Losartan inhibition of TGF-β signaling might reduce collagen deposition in the cornea. Myofibroblasts are activated fibroblasts that express alpha-smooth muscle actin (α-SMA) and are key effectors in fibrosis. Losartan modulates myofibroblast differentiation and activity, potentially limiting their presence in the cornea.

Furthermore, losartan demonstrated anti-inflammatory effects in various tissues. In corneal fibrosis, where inflammation often accompanies fibrotic processes, losartan might attenuate the inflammatory response. Maintaining corneal transparency is essential for vision. Therefore, the potential antifibrotic effects of losartan might preserve corneal transparency by preventing excessive scarring. Losartan has been investigated for its effect on wound healing. Controlled and balanced wound healing in the cornea is crucial to prevent excessive fibrotic reactions, and losartan might modulate this process [90,91,92].

Decorin is a small leucine-rich proteoglycan in the ECM of various tissues, including the cornea, and modulates collagen fibrillogenesis and influences cell behavior. Decorin has been studied for its potential antifibrotic effects, including its influence on corneal fibrosis. Decorin exerts its antifibrotic effects through various mechanisms, primarily by modulating TGF-β activity. A central mechanism by which decorin inhibits fibrosis is its ability to bind and sequester TGF-β. Decorin has a high affinity for TGF-β and can effectively sequester it, preventing its interaction with cell surface receptors. TGF-β is a potent fibrosis inducer, promoting fibroblast activation and ECM component synthesis.

Decorin inhibits the activation of fibroblasts, key cells producing collagen, and other ECM proteins. Activated fibroblasts are central in fibrosis development, and decorin maintains the quiescent state of these cells. Myofibroblasts are contractile cells characterized by α-SMA expression and contribute to tissue contraction and fibrosis. Decorin reduces fibroblast differentiation into myofibroblasts, limiting the cellular effectors responsible for excessive matrix deposition. Furthermore, decorin directly interacts with collagen types I and III, influencing collagen fibril assembly and organization. Decorin regulation of collagen fibrillogenesis maintains the proper ECM structure and composition, preventing dense fibrotic tissue formation.

Decorin exhibits anti-inflammatory properties that contribute to its antifibrotic effects. Inflammation is often associated with fibrosis, and decorin modulates the inflammatory response, reducing the overall fibrotic process. Decorin promotes tissue remodeling by influencing ECM turnover, enhances the degradation of existing matrix components, and facilitates balanced new matrix protein synthesis. Decorin is crucial for maintaining cornea transparency. Decorin regulation of collagen fibrillogenesis ensures proper collagen fiber organization in the cornea, contributing to its transparency.

Decorin also contributes to maintaining tissue homeostasis, where it regulates the balance between matrix synthesis and degradation, preventing excessive scar tissue accumulation. Decorin induces fibrotic cell apoptosis, contributing to the removal of cells that would otherwise contribute to fibrosis. Furthermore, decorin has been studied for its ability to modulate the corneal wound healing response. Controlled and balanced wound healing is essential to prevent excessive scar formation and fibrosis in the cornea. Due to its antifibrotic properties, decorin has been explored as a potential therapeutic agent for corneal fibrosis and other fibrotic disorders. In vitro and animal model studies have reported promising results regarding the ability of decorin to mitigate fibrotic responses. Gene therapy approaches to enhance decorin expression in the cornea have been investigated to augment the natural antifibrotic properties of decorin and limit fibrosis.

The multifaceted mechanisms of decorin highlight its potential as a therapeutic agent for fibrotic disorders, including those affecting the cornea, skin, liver, and other organs. As research in this field progresses, it might yield further insights into the detailed molecular mechanisms of its antifibrotic actions [93,94,95,96,97,98,99].

Pirfenidone is an antifibrotic and anti-inflammatory medication that has been studied in various fibrotic disorders, particularly pulmonary fibrosis. Pirfenidone has antifibrotic and anti-inflammatory properties, but its efficacy and safety in treating corneal fibrosis may require further investigation. While the exact mechanism of action of pirfenidone is not fully understood, it is believed to involve multiple pathways. TGF-β is a key cytokine in fibrosis, promoting scar tissue formation by stimulating ECM protein production. Pirfenidone is thought to inhibit TGF-β synthesis, reducing its fibrogenic effects. Chronic inflammation is often associated with fibrosis, and pirfenidone might indirectly reduce fibrotic processes by suppressing inflammation.

Pirfenidone is believed to interfere with the activity of fibroblasts, which produce collagen and other ECM components. Pirfenidone might prevent excessive scarring by inhibiting fibroblast proliferation and collagen synthesis [100,101,102,103]. Pirfenidone nanoparticles improve corneal wound healing and prevent fibrosis. Pirfenidone nanoparticles have potential in the treatment of corneal chemical burns and other fibrotic corneal diseases [104].

Dixon et al. hypothesized that the long-term delivery of the anti-inflammatory and antifibrotic drug pirfenidone through contact lenses containing vitamin E is an effective method of counteracting these processes. One week after an alkaline burn, eyes treated with pirfenidone contact lenses showed significant improvement in corneal opacity compared with control eyes. About 50% of the drug placed in the lens reached the aqueous humor compared to 1.3% for eye drops. Positive preclinical results indicate that this is a promising therapy for the treatment of corneal inflammation and fibrosis. The bioavailability is approximately 40 times higher for contact lenses compared to eye drops. Vitamin E-containing contact lenses serve as a suitable delivery platform for pirfenidone after alkaline burns in rabbit eyes and may also provide an effective anti-inflammatory and anti-fibrotic therapy in the future [105].

Pirfenidone has antioxidant effects, which might mitigate oxidative stress. Oxidative stress is implicated in fibrosis pathogenesis, and pirfenidone might prevent or impede fibrotic changes by reducing oxidative damage.

Notably, the precise mechanisms of pirfenidone’s action vary depending on the specific tissue or organ being studied. While pirfenidone has been approved for treating idiopathic pulmonary fibrosis (IPF), its applications in other fibrotic conditions, including corneal fibrosis or liver fibrosis, are undergoing research [100,101,102,103,106].

### 4.6. Ocular Nanosystems

Ocular nanosystem technology uses nanoparticles or nanomaterials to improve drug delivery to the eye. Nanoparticles allow for an increased efficiency and precision in delivering active ingredients to the eye. Ocular nanosystems can be used to treat eye conditions, including corneal disorders, cataracts, and retinal diseases. Nanotechnology allows drugs to be delivered to specific areas of the eye, which improves the treatment effectiveness and reduces possible side effects. Ocular nanosystems include nanosuspensions, liposomes, polymer nanoparticles, hybrid nanoparticles, biological nanomaterials, oil nanodroplets, dendrimers, gold nanoparticles, and nanopigments [106].

Some nanosystems may contain nanoparticle anti-VEGF drugs, which are nanomaterials containing VEGF inhibitors to treat eye diseases, especially those associated with uncontrolled angiogenesis.

Ranibizumab oil nanodrops are oil droplets used to improve ranibizumab penetration through the layers of the eye. Nanosuspensions are nanoparticles suspended in a liquid that can be used as eye drops and ensure an even distribution of the active ingredient on the eye surface. Antibiotic nanosuspensions are used to treat infectious eye conditions. Hybrid nanoparticles are combinations of different materials, such as polymers and metallic nanoparticles, that can combine different features, such as the ability to release drugs and image eye structures. Biological nanomaterials use biological nanoparticles, such as proteins or lipids, which minimizes the risk of allergic reactions and improves biocompatibility [106,107,108,109,110].

Novel ocular nanosystems based on nanotechnology constitute a new path in the treatment of corneal neovascularization. Compared with traditional drug delivery, ocular nanosystems can not only optimize penetration and bioavailability but also extend the drug retention time, thereby achieving sustained delivery and controlled therapeutic release with minimal toxicity and side effects. Some inorganic nanomaterials, such as AuNPs, AgNPs, CeNPs and carbon-based nanomaterials, are themselves antiangiogenic nanoagents. Nanotoxicity depends largely on various properties such as the material composition, concentration, biodistribution, size, shape, surface charge, and attached chemical groups. For example, positively charged biopolymers containing nanopreparations extend the retention time of the drug on the ocular surface, which may cause corneal toxicity. In turn, nanopreparations with too high a surfactant concentration may cause corneal damage. In some cases, the presence of surfactants may cause a sticky feeling and blurred vision after instillation [111].

Chang et al. reported the preparation of EGCG (epigallocatechin-3-gallate)-loaded nanoparticles using an arginine–glycine–aspartic acid peptide with a hyaluronic acid-conjugated complex coating gelatin. EGCG, which has anti-angiogenetic activity, was selected as an inhibitor for the treatment of corneal neovascularization. In this study, a mouse model of corneal neovascularization was used to evaluate the therapeutic effect of the applied nanoparticle in the form of eye drops. This study indicated that gelatin/EGCG nanoparticles (GEH-RGD) could be used in eye drops to inhibit angiogenesis in corneal neovascularization in mice [112].

Ocular nanosystems in the treatment of corneal neovascularization are still at the stage of preliminary research. Therefore, appropriately designed clinical trials for various ocular nanosystems in the treatment of corneal neovascularization are needed in the future [111].

### 4.7. Small Interfering RNA (siRNA) Gene Therapy

Corneal neovascularization involves excessive blood vessel growth, leading to visual impairment and even blindness. Blindness caused by corneal neovascularization is a permanent and challenging condition with limited treatment options. siRNAs block gene expression by targeting a specific mRNA, inhibiting the production of the encoded proteins. siRNAs might be a potential therapeutic strategy in corneal neovascularization, blocking the expression of angiogenesis-related genes. Several genes are involved in regulating neoangiogenesis. The potential applications of siRNAs in corneal neovascularization include blocking VEGF synthesis and regulating other genes associated with neovascularization.

siRNAs are a promising strategy for preventing corneal neovascularization. A novel approach used VEGFA siRNA (siVEGFA) to reduce VEGFA expression. A pH-sensitive polycationic mPEG(2k)-PAMA(30)-P(DEA(29)-D5A(29) (TPPA) was synthesized to improve siVEGFA delivery efficiency. The TPPA–siVEGFA polyplexes entered cells via clathrin-mediated endocytosis. Hemolytic assays confirmed that TPPA was safe in normal physiological environments (pH 7.4) but could easily damage the membranes of mature acidic endosomes (pH 4.0). In vivo distribution studies demonstrated that TPPA prolonged the siVEGFA retention time and promoted its penetration into the cornea. TPPA effectively delivered siVEGFA to the lesion site in a mouse model with induced alkali burns and reduced VEGFA expression. The inhibitory effect of TPPA–siVEGFA on corneal neovascularization was comparable to that of ranibizumab. Delivering siRNA to the ocular environment via pH-sensitive polycations provides a novel strategy for effectively inhibiting corneal neovascularization [113]. siRNAs can be engineered to target other key genes responsible for producing angiogenesis-related factors, such as *PDGF* and *FGF*. siRNA targeting of VEGFA, PDGF, and FGF can effectively inhibit angiogenesis [114,115,116,117,118,119,120,121,122,123,124].

Gene therapy for corneal neovascularization is an area of intense research. Gene therapy introduces modified genetic material to regulate the expression of specific genes associated with neovascularization processes. Potential gene therapy for corneal neovascularization might include the introduction of genes encoding inhibitors of vascular growth factors, such as endostatin or angiostatin, inhibiting the growth of new blood vessels (antiangiogenic gene therapy), modifying the expression of genes encoding vascular growth factors to control the neovascularization process, modulating the immune response to regulate inflammatory processes associated with corneal neovascularization, and promoting endothelial cell regeneration by introducing genes that promote endothelial cell regeneration to restore the normal structure and function of blood vessels in the cornea [125,126,127,128,129,130,131]. Corneal neovascularization is a common clinical symptom of many corneal diseases. Current therapeutic approaches to the treatment of corneal angiogenesis, in which VEGFA plays a major role, may cause a variety of adverse side effects. Clustered Regularly Interspaced Short Palindromic Repeat (CRISPR/Cas9) technology allows for editing of the *VEGFA* gene to suppress its expression. Zeng et al. demonstrated that depletion of VEGF A using a novel CRISPR/Cas9 system inhibits the proliferation, migration, and tube formation of human umbilical vein endothelial cells (HUVECs) in vitro. Subconjunctival injection of this dual AAV-SpCas9/sgRNA-VEGFA system blocks suture-induced VEGFA expression, CD31, and α-smooth muscle actin, as well as corneal neovascularization in mice. CRISPR offers a new option for the treatment of corneal neovascularization. The authors of this study claim that it has created a strong basis for the novel treatment of corneal neovascularization using the gene editing method to correct genetic defects [132].

Gene therapy targeting VEGF has yielded positive results. An adenoviral vector carrying *VEGFR* genes causes corneal neovascularization regression. When injected subconjunctivally in a rat model, the vector effectively inhibited corneal neovascularization. A viral vector containing the human angiostatin gene (a protein angiogenesis inhibitor) exerted similar inhibitory effects. Although gene therapy has demonstrated promising efficacy, technical issues limit its widespread use [2].

### 4.8. Antisense Oligonucleotides

Aganirsen works through an antisense mechanism, interfering with the synthesis of specific proteins by binding to the corresponding mRNA. Aganirsen is designed to target insulin receptor substrate-1 (IRS-1) associated with angiogenesis. IRS-1 is vital in neovascularization and is overexpressed in corneal angiogenesis. Aganirsen dose-dependently inhibits IRS-1 expression and angiogenesis while reducing VEGFA and IL-1β mRNA levels [133,134,135].

Aganirsen has undergone clinical trials to evaluate its safety and efficacy in treating corneal neovascularization. Clinical trial results may vary, and the underlying cause of neovascularization and individual patient characteristics might influence aganirsen effectiveness in corneal neovascularization. Aganirsen is not primarily known as a PDGF receptor inhibitor. While the insulin growth factor-1 (IGF-1) signaling pathway, which includes IRS-1, may interact with various growth factors, including PDGF, the aganirsen mechanism of action is targeted towards IRS-1. PDGF receptor inhibitors, such as imatinib, sunitinib, pazopanib, directly target PDGF receptors, which are part of the PDGF signaling pathway [136].

Aganirsen in the form of squalamine lactate eye drops has been studied for its potential in treating corneal neovascularization. Topical aganirsen application was well tolerated and caused no side effects. After 90 days, the corneal neovascularization area was reduced by 26.2%, and the improvement persisted after 180 days [136,137].

### 4.9. Other Potential Therapies

#### 4.9.1. TIMPs

TIMPs are endogenous inhibitors produced by cells to regulate MMP activity by binding to active MMPs and inhibiting their enzymatic function. There are four known TIMPs (TIMP1–4), which have similar structures but differing degrees of affinity and expression profiles. Cytokines and growth factors regulate TIMP activity. TIMP2–4 inhibit all known human ECM proteases. Doxycycline is a tetracycline group antibiotic that is a non-selective inhibitor of metalloproteinases. Doxycycline inhibits metalloproteinase activity by chelating zinc and calcium ions. In inflammatory conditions, suppressing enzymes that compromise the corneal structural integrity can block corneal neovascularization. Oral doxycycline is used at a dose of 100 mg twice daily on the first day of corneal neovascularization treatment, followed by a maintenance dose of 100 mg once daily. Combining orally administered doxycycline with topical corticosteroids inhibits neovascularization [44].

#### 4.9.2. TGF-β Inhibitors

TGF-β inhibitors are substances or drugs designed to block TGF-β activity. TGF-β is a multifunctional cytokine crucial to cellular processes, including cell growth, differentiation, and immune responses. An increased TGF-β1 activity is implicated in ECM synthesis and remodeling and progressive fibrosis [93]. TGF-β signaling dysregulation has been observed in several diseases, including cancer, fibrosis, and autoimmune disorders. Table 4 presents some examples of TGF-β inhibitors.

TGF-β inhibitors in clinical settings are an area of active research, and the development of effective and safe inhibitors is ongoing. Inhibiting TGF-β signaling has been a research focus for potential therapeutic interventions in fibrotic diseases. TGF-β has complex and context-dependent functions; therefore, therapeutic interventions targeting this pathway should carefully consider the potential side effects and overall effect on the immune system and tissue homeostasis. TGF-β inhibitors have been explored for their potential to prevent or impede fibrosis progression in tissues and organs, including the liver, lungs, kidneys, heart, and cornea. Some approaches involve combining TGF-β inhibitors with other antifibrotic agents or standard treatments to mitigate fibrosis synergistically [138,139,140].

TGF-β is also involved in corneal fibrosis. Inhibiting TGF-β signaling has been explored as a potential therapeutic approach to mitigate corneal fibrosis. TGF-β is a potent fibroblast activator, and dysregulated TGF-β signaling transforms quiescent fibroblasts into myofibroblasts, responsible for excessive matrix deposition. TGF-β inhibitors aim to reduce fibroblast activation in the cornea. TGF-β stimulates the synthesis of collagen types I and III, major ECM components. TGF-β inhibitors inhibit collagen production, preventing excessive collagen fiber buildup in the cornea. Myofibroblasts, which express α-SMA, are key effectors in fibrosis. TGF-β inhibitors target corneal fibroblast differentiation into myofibroblasts, preventing their accumulation.

TGF-β inhibitors might have anti-inflammatory effects on the cornea, contributing to an overall reduction in fibrotic responses. A critical aspect of corneal health is its transparency. Corneal fibrosis disrupts clarity, impairing vision. TGF-β inhibitors aim to preserve corneal transparency by preventing excessive scarring. TGF-β inhibitors might promote more controlled and balanced wound healing in the cornea, facilitating a regenerative response rather than an excessive fibrotic reaction.

Research in this area is ongoing, and the development of specific TGF-β inhibitors for corneal fibrosis is an active area of investigation. Some studies have focused on TGF-β-neutralizing antibodies, small-molecule inhibitors, and other strategies to modulate TGF-β signaling in the cornea. However, TGF-β has diverse functions in tissue homeostasis and repair, and complete inhibition might have unintended consequences [141,142,143,144,145]. Therefore, the safety and efficacy of any therapeutic approach targeting TGF-β must be carefully evaluated.

#### 4.9.3. IL-1 Receptor Antagonist (IL-1 Ra)

IL-1 Ra is a naturally occurring protein that regulates various tissue inflammatory responses. IL-1 Ra belongs to the IL-1 family of proteins and is currently used in rheumatoid conditions as an anti-inflammatory drug, inhibiting IL-1α and IL-1β biological activity. IL-1 Ra is produced under the influence of cytokines, viral antigens, and acute-phase proteins and is a limiting factor for IL-1β in the chronic inflammation phase. IL-1 Ra is promising for treating corneal neovascularization.

IL-1 binds to two receptor types: IL-1 type 1 receptor (IL-1RI) and IL-1 type 2 receptor (IL-1 RII) [146]. Stimulation of IL-1RI involving an additional IL-1RAcP protein induces signal transduction and action depending on the cell type undergoing activation. Binding to IL-1RII does not lead to cell signaling. IL-1Ra has been studied for its potential in treating corneal neovascularization due to its ability to modulate the inflammatory response. IL-1Ra binds to the IL-1 receptor and prevents the proinflammatory effects of IL-1, including the expression of proangiogenic molecules such as VEGF and inducible NO synthase (iNOS). IL-1 Ra reduces inflammation and potentially inhibits new blood vessel growth in the cornea by blocking the action of IL-1 (IL-1α and IL-1β) [1,22]. IL-1Ra eye drops (2.5%) were effective and well tolerated in treating epitheliopathy [147].

#### 4.9.4. Rho Kinase Inhibitors

Rho-related protein kinase (ROCK) is a serine/threonine kinase that regulates various cellular processes. ROCK inhibitors are well established in the treatment of glaucoma, but also have therapeutic potential in the treatment of corneal diseases by increasing cell proliferation and adhesion and reducing apoptosis.

Among ROCK inhibitors, four have been confirmed to be effective for the treatment of corneal diseases: Y-27632, fasudil, ripasudil, and netarsudil. In Fuchs’ corneal endothelial dystrophy, Y-27632, ripasudil, and netarsudil led to improvement in corneal clarity, endothelial cell density, and visual acuity. In bullous keratopathy, the injection of Y-27632 together with cultured corneal endothelial cells into the anterior chamber leads to an increase in corneal endothelial cell density and an improvement in visual acuity. In endothelial iris corneal syndrome, netarsudil reduces corneal edema. After the use of fasudil, a significant reduction in corneal neovascularization, a reduction in corneal epithelial defects, and a reduction in inflammatory infiltrates were observed in an animal model after chemical corneal damage. Fasudil is a dual ROCK-1/2 inhibitor, formerly known as HA-1077; its active metabolite is hydroxyfasudil, a ROCK inhibitor that has been clinically approved for the treatment of cerebral vasospasm in Japan. However, fasudil has a low in vitro potency, and therefore its clinical use in ophthalmology remains limited [148].

Zeng et al. showed that the ROCK inhibitor fasudil showed significant efficacy in inhibiting corneal neovascularization in mice after alkali burns. The administration of 100 μM fasudil eye drops four times daily led to a marked reduction in the incidence of neovascularization and was correlated with reduced inflammatory cell infiltration, reduced production of reactive oxygen species, and increased heme oxygenase-1 activity. Acceleration of the healing of corneal epithelial damage was also observed [148,149].

The rho-related protein kinase (ROCK) inhibitor AMA0526 was evaluated for its effects on corneal neovascularization and scarring in various in vitro and in vivo experimental models. The inhibitor AMA0526 significantly inhibited vascular endothelial cell proliferation and migration in vitro in a dose-dependent manner. Corneal neovascularization was reduced by 37%, inflammatory infiltrates were reduced by 26%, angiogenesis was reduced by 47%, and collagen III deposition was reduced by 27%. The administration of AMA0526 showed similar effects to dexamethasone with additional antifibrotic effects. These results justify the need for further research into the therapeutic potential of AMA0526 in the treatment of corneal neovascularization and scarring [150].

By examining the safety profiles of ophthalmic ROCK inhibitors, both netarsudil and ripasudil have been approved for clinical use, supported by numerous studies. However, limited data exist on the safety profile of fasudil, which has been approved only for the treatment of cerebral vasospasm. ROCK inhibitors show promise in the treatment of corneal diseases. Analysis of their pharmacological properties and studies confirming their effectiveness emphasize their potential therapeutic importance [148].

#### 4.9.5. Sonic Hedgehog Protein

Insufficient, impaired, or excessive healing of corneal wounds creates major problems in therapy. There is increasing evidence for the role of the Hedgehog signaling pathway in corneal wound healing. The Sonic Hedgehog protein (Shh) is involved in numerous processes of cellular or tissue homeostasis and oxidative stress. Hedgehog signaling and its effectors primarily participate in corneal wound healing at the level of the corneal epithelium and corneal limbus, where Sonic Hedgehog-mediated signaling promotes limbal stem cell proliferation and corneal epithelial cell proliferation and migration following corneal injury. Hedgehog signaling may also participate in corneal epithelial barrier homeostasis and in pathological corneal healing, such as neovascularization [151].

The effect of Shh on the expression of angiogenic cytokines and its receptor Patched 1 (Ptc) was studied in cultured cells in a vascularized mouse cornea after alkaline burns. The effect of exogenous Shh, VEGF, and/or the Shh signal inhibitor cyclopamine on the formation of corneal neovascularization was also investigated. The addition of Shh promoted the formation of new blood vessels, which was inhibited by cyclopamine. Shh and Ptc mRNA and protein expression levels were below the limit of detection in the intact cornea, while levels of Shh, but not Ptc, were elevated in the healing, vascularized cornea [152].

#### 4.9.6. Tyrosine Kinase Inhibitors

VEGF exerts its angiogenic effects through two major receptor tyrosine kinases (RTKs), VEGFR-1 (flt-1) and VEGFR-2 (flk-1/KDR), which are expressed mainly by vascular endothelial cells. Although VEGFR-1 has a higher affinity for VEGF, its activity is much weaker than VEGFR-2. On the other hand, VEGFR-1 is negatively regulated in the cornea by sflt-1 and a low-affinity VEGF isoform called VEGF165b that competes with VEGFA. The angiogenic activity of VEGF is mainly associated with the VEGFR-2 pathway, whereas VEGFR-1 has regulatory rather than angiogenic effects [153].

- Sunitinib

Sunitinib is a multitargeted receptor tyrosine kinase (RTK) inhibitor that selectively and potently inhibits four RTK receptors involved in angiogenesis: VEGFR-2, PDGFR-β, FGFR-1, and EGFR. Oral administration of sunitinib in inflammation-induced corneal neovascularization in mice effectively reduces angiogenesis, most likely by blocking the VEGFA/VEGFR-2 pathway [154]. Topical administration of 0.5 mg/mL sunitinib has also been associated with significant reductions in VEGFR-2 levels and neovascularization. Topically administered sunitinib is three times more effective than bevacizumab in treating corneal neovascularization due to its inhibitory effects on VEGF and PDGF pathways. Furthermore, better results were obtained after local administration compared to subconjunctival injection [155]. The safe dose of topical sunitinib has not yet been established, and toxic effects on the corneal epithelium have been observed at concentrations greater than 3.3 µg/mL [153,156].

- Lapatinib

Lapatinib blocks two RTK receptors, including human epidermal growth factor receptor 2 (HER2) and epidermal growth factor receptor (EGFR). Oral administration of lapatinib effectively reduces corneal neovascularization by reducing VEGF expression in the corneal epithelium and corneal stroma in an animal model [153,157].

#### 4.9.7. NOS

There are three isoforms of NOS: endothelial (eNOS or NOS1), neuronal (nNOS or NOS2), and inducible (iNOS or NOS3) isoforms. The role of NOS in corneal neovascularization is complex. Endothelium-derived NOS is found in vascular endothelial cells and is critical for regulating vascular tone and blood flow. NOS promotes angiogenesis by stimulating the proliferation and migration of endothelial cells, which are essential for new blood vessel formation. NOS might also have antiangiogenic effects by inhibiting VEGF-mediated signaling, a major angiogenesis driver.

nNOS is mainly found in nerve cells and is involved in neurotransmission. Its role in corneal neovascularization is unclear, and its involvement might be limited compared to other isoforms. iNOS is typically not expressed in healthy tissues but can be induced by proinflammatory factors such as cytokines and bacterial endotoxins. iNOS-derived NO often contributes to inflammation and immune responses. iNOS expression might promote angiogenesis by recruiting immune cells and facilitating tissue remodeling.

Interactions between NOS isoforms and their effect on corneal neovascularization might vary with the underlying causes of neovascularization and the overall inflammatory microenvironment. Additionally, the balance between NO’s proangiogenic and antiangiogenic effects complicates understanding of its role. Research into the molecular processes involved in corneal neovascularization is ongoing, and potential therapeutic strategies targeting NOS signaling are being developed [158].

#### 4.9.8. Galectin-3 Inhibitors

Anti-VEGF drugs are therapeutically successful. However, their effect is limited against mature neovascularization, which involves pericyte-covered vessels. Galectin-3 inhibitors appear promising as they attenuate the corneal neovascularization course and affect fibrotic processes in the cornea [159].

#### 4.9.9. PEDF

PEDF inhibits angiogenesis via VEGF, bFGF, and IL-8 (CXCL8) by inhibiting endothelial cell migration to neovascularization areas and concomitantly inducing apoptosis [160]. Exogenous PEDF might inhibit new blood vessel growth and reduce corneal neovascularization [161].

#### 4.9.10. PDGF Receptor Inhibitor

PDGF is a protein that is significant in cell growth and division. PDGF is released by platelets and cells involved in blood clotting, wound healing, and tissue repair processes. PDGF has proangiogenic and mitogenic effects and interacts with other growth factors and cytokines. This interaction can enhance the angiogenic response and contribute to new blood vessel development. Intraperitoneal injection of a PDGF receptor inhibitor reduced pericapillary pericytes and vascular density in advanced corneal neovascularization [162].

## 5. Surgical Treatment

### 5.1. Fine Needle Diathermy (FND)

FND is commonly used to occlude corneal vessels and is performed using a 3/8 needle with a 10-0 nylon suture [163,164]. FND involves inserting the needle close to the limbus parallel to the vessel and at the same depth as the vessel to be closed. Inserting the needle tip into the vessel lumen is possible with larger vessels. Diathermy is continued until mild whitening of the corneal stroma is observed. Then, the procedure is repeated in other areas affected by neovascularization. FND can be performed simultaneously with corneal transplantation, reducing the risk of intraoperative bleeding and graft rejection [165]. Needle diathermy complications are reversible and include intracorneal stromal hemorrhages, transient corneal opacity, transient corneal whitening in the stroma adjacent to the needle, which completely resolves after 24–48 h, and microperforations, especially with thin corneas [2]. FND heat energy might have adverse effects on limbal stem cells and the endothelium. FND might also secondarily lead to neovascularization due to the release of proangiogenic substances. A procedure modification was introduced to reduce FND-related complications, where diathermy is performed under fluorescein angiography guidance [165,166].

FND is a promising surgical procedure for the treatment of corneal neovascularization. Various studies have shown that the treatment is an effective and safe method of occluding pathological vessels. FND also possibly reverses the rejection of a corneal transplant. Some patients benefit from a single procedure, while others benefit from a second procedure; however, some patients did not gain a response after three attempts. FND can be combined with topical anti-VEGF treatment, which may increase the effectiveness of the procedure. To summarize, FND is a safe and effective procedure in selected patients, but may not be effective [167].

### 5.2. Laser Therapy

Laser therapies (argon laser photocoagulation and a 577 nm yellow laser) can be used to obliterate abnormal blood vessels in the cornea, preventing transplanted flap rejection, and are used in treating lipid keratopathy. Laser photocoagulation facilitates the obliteration of large-diameter and slower-blood-flow efferent vessels. Contrastingly, it is difficult to obliterate afferent vessels as they are thinner, more deeply located in the cornea, and characterized by a rapid blood flow. In this case, laser therapy is ineffective, and thermal damage due to repeated use might promote neovascularization recurrence [168,169]. The complications of laser therapy can include corneal endothelial damage, corneal thinning, intraocular hemorrhage, pupillary displacement, iris atrophy, and corneal suture lysis after corneal transplantation [2].

Photodynamic therapy (PDT) is not the standard treatment for corneal neovascularization; its use is more well established in treating choroidal neovascularization. This minimally invasive treatment eradicates the pathological corneal vascular network without damaging healthy tissue. PDT generates reactive oxygen species that destroy the endothelium and vascular basement membrane, causing local thrombosis and vascular remodeling. An experimental study administered intrastromal injections of verteporfin, a photosensitizing drug before PDT, and reported selective regression of lymphatic vessels [170]. PDT with verteporfin and intrastromal bevacizumab injection effectively inhibited corneal neovascularization in patients with Stevens–Johnson syndrome [171]. PDT has minimal systemic effects, making it safe when multiple sessions are needed. PDT is a feasible option for patients with visual impairment caused by lipid keratopathy secondary to corneal neovascularization. Testing PDT efficacy for corneal neovascularization led to good results and safety at 6–24 months [172,173,174]. We should remember that PDT is not a standard form of therapy and not every pathological corneal vessel will be effectively obliterated.

Nd:YAG laser photocoagulation (532 nm) can reduce the corneal neovascularization area without significant side effects. Three months after the completion of frequency-doubled Nd:YAG (532 nm) laser photocoagulation, the corneal opacity area and corneal neovascularization area reductions were 7.01% and 44.08%, respectively. Dual-frequency Nd:YAG (532 nm) laser photocoagulation has proven to be a safe and effective method of treating corneal vascularization, demonstrating an effective reduction in the area of corneal vascularization. However, 37.29% of the vessels were recanalized 3 months after the procedure, suggested repeated laser treatments might be needed in some patients [175].

A femtosecond laser appears to be effective for treating corneal neovascularization. Nevertheless, studies have only been conducted on an animal model. Furthermore, this treatment method is not widely used given the high equipment cost [2].

### 5.3. Amniotic Membrane Transplantation

The amniotic membrane consists of an epithelium monolayer, a thick basement membrane, and a non-vascularized, hypocellular matrix. The amniotic membrane releases TIMP2 proteins that significantly reduce bFGF and inhibit fibroblast differentiation into myofibroblasts. Corneal neovascularization treatment might involve suturing the amniotic membrane, which has anti-inflammatory and antiangiogenic effects [166].

The amniotic membrane is ideal for supporting corneal epithelial proliferation. Laminin, fibronectin, type IV, V, and VII collagen, and integrins 4 and 6 in the amniotic membrane stimulate the differentiation and hyperplasia of corneal epithelial cells, increase their adhesion to the basement membrane, and replenish limbal stem cells. The amniotic basement membrane composition is similar to the conjunctiva and cornea basement membrane, making it an almost ideal material for reconstructing the ocular surface. Yin and Pi have shown that in patients with a chemical alkali burn of the cornea, amniotic membrane transplantation can inhibit the development of corneal neovascularization. In their study, on the 14th day after the burn, the area of neovascularization was 18.27% smaller than in the control group, and on the 60th day, it was 11.35% smaller. In both cases, the results were statistically significant. The amniotic membrane may inhibit the growth of fibrovascular tissue and the formation of neovascularization. These results suggest that amniotic membrane transplantation can inhibit the development of corneal neovascularization, which may be beneficial for the treatment of early-stage severe alkaline eye burns [176,177]. Amniotic membrane transplantation is a supportive treatment, and corneal neovascularization should be treated using multiple modalities.

### 5.4. Superficial Keratectomy

Superficial keratectomy surgically removes the superficial layers of the cornea, i.e., the epithelium, Bowman’s membrane, and occasionally the superficial corneal stroma layers. Superficial keratectomy can be used to treat various corneal conditions, including neovascularization. Superficial keratectomy is targeted at removing abnormal blood vessels that reduce corneal translucency and impair corneal function, leading to corneal scarring, inflammation, decreased visual acuity, and even vision loss. Intraoperatively, 0.02% mitomycin C can be used to reduce postoperative corneal haze or amniotic membrane transplantation to improve surgical outcomes. Superficial keratectomy eliminates the underlying cause, i.e., abnormal blood vessels, stimulating the corneal natural healing processes. After the procedure, the cornea begins to heal, and the surrounding healthy tissue regenerates.

Corneal neovascularization after superficial keratectomy and subconjunctival injection of bevacizumab (2.5 mg/0.1 mL) regressed and showed no signs of recurrence after 3 months of follow-up. This combination therapy may represent a treatment strategy for superficial corneal neovascularization.

Superficial keratectomy is often combined with other treatments, such as topical or laser therapy. These additional treatments control the underlying condition that caused the neovascularization. While superficial keratectomy can be effective in treating corneal neovascularization, its success depends on the cause of the neovascularization, the degree of blood vessel growth, and the patient’s overall eye condition. Additionally, superficial keratectomy might involve complications, such as infection, delayed healing, or neovascularization recurrence [178]. Superficial keratectomy is a possible treatment option for corneal neovascularization but has potentially serious complications.

### 5.5. Corneal Transplantation

Corneal transplantation is performed in severe cases where neovascularization has significantly impaired visual acuity. Patients with corneal neovascularization are prepared for corneal or corneal limbus transplantation by subconjunctival injection of anti-VEGF solution. Antiangiogenic treatment is also administered after corneal transplantation to prevent transplanted flap rejection and continues after corneal limbus transplantation until a stable epithelium is obtained [166].

Immune privilege is observed in some tissues that exhibit a limited immune response. The cornea is such a tissue with several characteristics that make it less prone to immune responses. The cornea is devoid of blood and lymphatic vessels, which limits immune cell access to potential antigens and is a protective factor against allograft rejection. Ocular surface trauma or infection can induce corneal neovascularization, a significant risk factor for corneal allograft rejection. Furthermore, the new vessel ingrowth causes the loss of immune privilege and increases the graft rejection risk [170,178].

The small number of T lymphocytes in the cornea renders immune responses less likely. Immunomodulatory factors such as growth factors and cytokines are present in the cornea, which maintains immune tolerance. The cornea can maintain an anergic immune state when proangiogenic and antiangiogenic factors are balanced (corneal angiogenic privilege), which means that the immune cells in its surroundings are more likely to be tolerant. In corneal transplantation, the immune privilege of the cornea does not preclude the transplant rejection risk, which can occur especially if the immune system cells identify the transplanted corneal cells as foreign and initiate an immune response [153,179].

Corneal transplantation plays a key role in the treatment of corneal blindness. For uncomplicated corneal transplants first performed in avascular hosts (i.e., normal risk transplants), graft survival rates often exceed 90%. However, grafts from hosts with inflammation and vascularized host beds are at increased risk of immune rejection, which may occur in >50% of high-risk grafts [180]. The rejection risk is reduced using immunological matching between the donor and recipient and immunosuppressive drugs, which weaken the immune response and maintain graft longevity [153,179].

Keratoprosthesis placement may be the treatment of choice for patients with repeated graft failure or rejection due to refractory central corneal neovascularization.

### 5.6. Corneal Limbal Stem Cell Transplantation

The cornea must be transparent to maintain its visual function and have a regular shape to maintain its refractive power. The transparency of the cornea is the result of collagen fibrils in the stroma that are devoid of blood vessels. Any corneal immune process can lead to corneal scarring and pathological angiogenesis. The corneal epithelium remains an immunologically active structure, characterized by the high activity of cytokines such as IL-1, IL-6, IL-8, and granulocyte-monocyte colony-stimulating factor (GM-CSF). The corneal limbus is the peripheral part of the cornea, located at the edge of the sclera within the palisades of Vogt structure, and contains stem cells. The presence of the limbal epithelium creates a barrier that prevents corneal conjunctivization. Limbal epithelial stem cells (LSCs, LESCs) are multipotent cells responsible for the reconstruction of the corneal epithelium used in corneal regenerative treatment in patients with limbal epithelial stem cell deficiency (LSCD, LESCD) [181].

The cellular phenotype of LSCs indicates the primary expression of p63, ABCG2 and α9 integrin proteins. Antibodies found in limbal cells include antibodies against β1 integrin, epidermal growth factor receptor (EGFR), K19, α-enolase, and CD71 [182]. The ATP-dependent transporters ABCG2 and ABCB5 are the most studied transporters found in LSCs [183]. Other molecules important for maintaining LSC function are C/EBPδ, Bmi-1, and p63α. Their role is to maintain the cell cycle, but they can also be used as a predictor of positive LSC transplantation outcomes. A high percentage of p63-positive cells in the LSC graft is a predictor of LSC graft success [184].

Limbal stem cell transplantation is a possible therapeutic tool in patients with limbal epithelial stem cell deficiency (LSCD, LESCD). LSCD can be the result of many inherited or acquired conditions, such as chemical or thermal damage to the cornea, contact lens keratopathy, and Stevens–Johnson syndrome. These conditions cause opacification of the corneal epithelium, followed by opacity, inflammation, neovascularization, and eventual scarring of the cornea.

Methods of limbal stem cell transplantation include direct autologous transplants, where the graft is taken from the patient’s healthy eye; direct allogeneic transplant, where the graft is taken from a healthy donor; and cultured autologous or allogeneic grafts, where the graft is taken and expanded from LSC samples and the stem cells are obtained from the patient’s or donor’s eye [185].

The only effective treatment for this disease is limbal stem cell transplantation. Current surgical procedures used to treat LSCD include SLET (simple limbal epithelial transplantation corneally limbal), CLAU (conjunctival–limbal autografting), CLET (ex vivo cultivated limbal epithelial transplantation), and KLAL (keratolimbal allograft). Cells used for transplantation may come from the patient (autologous transplants) or from a donor (allogeneic transplants).

Simple limbal epithelial transplantation (SLET) can be used in patients with a unilateral limbal stem cell deficiency. Donor tissue is harvested from a 2 mm × 2 mm area of the limbus and the graft is then placed under the human amniotic membrane (HAM). There is also a modification of SLET (M-SLET), in which the stem cell transplant is placed between two layers of amniotic membranes, the second of which is sutured to the limbal area [186,187].

The conjunctival–limbal autografting (CLAU) kinase procedure is characterized by the transplantation of autologous limbal tissue from the healthy eye to the contralateral side. This method is used in cases of unilateral LSCD. Limbal grafts are taken from the 6 and 12 o’clock positions of the healthy eye. CLAU is most often used in surgery for pterygium or severe chemical burns of the cornea. Recent reports show the long-term stability of the ocular surface after CLAU. CLAU can effectively provide long-term ocular surface and visual stability in 77.8% of eyes in patients with unilateral LSCD. However, one limitation of this procedure is that approximately one-third of autologous limbal tissue from the healthy eye is required for CLAU, which may pose a potential risk of developing LSCD in the healthy eye. CLAU is the most effective form of limbal stem cell transplantation that maintains long-term ocular surface stability in unilateral LSCD. However, CLAU is not applicable in bilateral LSCD cases. In such cases, corneal and limbal allograft (keratolimbal allograft, KLAL) techniques and living-related conjunctival limbal allografts (lr-CLAL) are options for restoring the ocular surface in bilateral LSCD [182].

The keratolimbal allograft (KLAL) procedure uses cadaverous limbal tissue as a source of limbal stem cells. This technique uses two donor corneoscleral rims, transplanting a full 360° corneal limbus. This technique is reserved for patients with bilateral LSCD, for patients who do not have a willing living relative for Ir-CLAL, and for patients with unilateral disease who were hesitant about whether their single healthy eye could be a source of stem cells. Systemic, long-term immunosuppression is required to ensure long-term graft survival and achieve good outcomes. The risk of graft rejection in this method is very high. After a KLAL, side effects can be long term and frequent. These include anemia, hyperglycemia, elevated creatinine levels, and elevated levels of liver function markers [188].

Ex vivo cultured limbal epithelial grafts (CLET) are divided into autologous CLET (auto-CLET) and allogeneic CLET (allo-CLET), which have been used in clinical trials (249). Compared with CLAU, KLAL, and lr-CLAL, the advantage of CLET is that less donor tissue is required, with shorter corneal epithelialization times and a lower risk of graft rejection. Auto-CLET is considered an ideal surgical method for unilateral LSCD to avoid the risk of immune rejection. In bilateral cases of complete LSCD, an effective method is allo-CLET, which uses corneal limbal stem cells from a deceased donor to restore the corneal structure without the need for biopsy. There are several types of scaffolds for in vitro cell expansion, such as human amniotic membranes, fibrin matrixes, human anterior lens capsules, silk fibroin, and siloxane hydrogel contact lenses. The immune rejection rate is low, recorded as 4.76% and 23.8%, respectively, at 6 months and during a follow-up period of 17.8 ± 3.8 months after surgery [182].

The choice of stem cell transplantation for patients with LSCD is difficult and depends on the LSCD severity. When autologous transplantation is not possible, allogeneic transplantation is performed, as in the case of severe bilateral LSCD. The prognosis in allografts is much worse, even despite intensive immunosuppressive treatment.

## 6. Combination Therapies

Combination therapies exert synergistic effects and translate into a better therapeutic outcome. Combining anti-inflammatory drugs and antiangiogenic agents, e.g., dexamethasone with bevacizumab, is more effective for corneal neovascularization than monotherapy [189]. Combining bevacizumab with argon laser therapy markedly decreases corneal angiogenesis. Argon laser photocoagulation closes abnormal corneal vessels, while bevacizumab prevents neoangiogenesis [168,190].

Corneal neovascularization treatment usually requires an individualized approach, and the choice of specific drugs and methods depends on the disease cause and severity and the patient’s overall health. Some drug–method combinations that may be considered for treating corneal neovascularization include anti-VEGF + laser therapy (especially argon lasers), anti-VEGF + anti-inflammatory drugs (corticosteroids or NSAIDs if the neovascularization is associated with inflammation), anti-VEGF + causal treatment (autoimmune diseases, diabetes, or infection), anti-VEGF + photothermal therapy or PDT (photosensitizers and lasers), laser therapy + anti-inflammatory drugs, and multi-drug combinations. Consensus is still required for the ideal therapeutic approach for actively growing (newly formed and small vessels) and established corneal neovascularization (large-caliber and mature vessels).

The primary therapeutic strategy includes steroids and VEGF inhibitors as monotherapy, but some combination therapies have been investigated with promising results. The assumption is that the combined therapies will be more effective in inhibiting vessel growth progression. Combination therapy typically allows smaller doses of individual drugs, translating into better treatment tolerance and less severe side effects. An example of combination therapy used to treat corneal neovascularization includes a topical combination of triamcinolone acetonide (10 µg/mL) with doxycycline (10 mg/mL). Corticosteroids are conventionally referred to as angiostatic steroids (e.g., triamcinolone acetonide) and have indirect antiangiogenic effects through their anti-inflammatory properties. Angiostatic steroids are believed to modulate collagen metabolism, which can lead to a complete breakdown of the vascular basement membrane [41,42]. Such combinations exhibit synergistic effects and effectively inhibit neovascularization progression. Small-molecule heparins appear to modulate antiangiogenic and proangiogenic factor expression by reducing the binding of proangiogenic factors to their receptors [191]. Topically applied doxycycline had a complementary antiangiogenic effect [127,128].

Other combination therapies potentially more effective than monotherapy include the combined use of anti-inflammatory and antiangiogenic drugs, e.g., dexamethasone or etanercept with bevacizumab [129,130], and combining bevacizumab with argon laser photocoagulation, FND [134,135], and PDT [137], which significantly reduces corneal angiogenesis [133]. Argon lasers, FND, and PDT seal abnormal corneal vessels, while bevacizumab prevents neoangiogenesis. The numerous steroid side effects, including steroid-induced glaucoma and increased susceptibility to infection due to immunosuppression, require a careful evaluation of the benefit–risk balance [2].

## 7. Summary

Corneal neovascularization is a relatively common consequence of severe ocular surface and corneal pathologies, which can significantly impair vision. Vascular neovascularization results from a complex mechanism involving inflammation, hypoxia, innervation disorders, and limbal stem cell deficiency. Continuous advances in understanding new molecular pathways and diagnosing corneal neovascularization severity facilitate novel and better therapeutic strategies.

The treatment of corneal neovascularization is chronic, difficult, and does not always have the desired effect. The choice of treatment modality depends on the clinical condition, as often a single procedure is insufficient and combined therapies should be used.

The treatment options for corneal neovascularization remain dependent on the blood vessel maturity. The primary therapy for corneal neovascularization includes anti-inflammatory and anti-VEGF drugs. In addition to VEGF, angiogenesis is also promoted by PDGF, bFGF, NO, and proinflammatory cytokines. Therefore, combination therapies acting on multiple angiogenic pathways might be more effective. The best strategy in severe limbal stem cell deficiency with deep neovascularization is ocular surface stem cell transplantation (OSST), followed by keratoplasty. Patients with repeated graft failure or rejection may benefit from keratoprosthesis placement as the treatment of choice. There is considerable hope for gene therapy, which might become a universal treatment for pathological corneal vasculariszation, regardless of lesion severity.

New therapies and new means of precise drug delivery to the diseased site, especially ocular nanosystems, raise great hopes for improving treatment effectiveness. siRNAs have the potential to target multiple genes, and comprehensive clinical research in this field is progressing.

## Figures and Tables

**Table 1:** Factors predisposing a patient to ocular neovascularization.

Local Risk Factors	Systemic Diseases	Corneal Lymphangiogenesis
Inflammatory conditions (chronic corneal inflammation, such as in keratitis, most commonly herpetic or chlamydial, chronic conjunctivitis, and recurrent uveitis) Infections (severe corneal infections, such as bacterial, viral, or fungal infections, and onchocerciasis) Contact lens wear (prolonged use, particularly of soft hydrogel lenses, incorrect use, poor fitting causing reduced oxygen supply to the cornea) Corneal transplants, corneal grafts (neovascularization as a complication following corneal transplantation, mainly if graft rejection or inadequate healing occurs) Corneal dystrophies (some inherited corneal disorders, such as certain corneal dystrophies) Chemical injuries (exposure to certain chemicals or irritants causing cornea damage) Trauma (corneal injuries, whether blunt trauma or penetrating injuries that disrupt the typical corneal structure) Dry eye syndrome (severe dry eye that results in corneal damage and triggers neovascularization) Limbal stem cell deficiency (conditions that affect the limbal stem cells, which maintain the health of the cornea)	Diabetes (impaired corneal sensation, corneal epithelial changes, chronic inflammation, altered corneal metabolism, reduced oxygen supply) Chronic allergic reactions (inflammatory response, compromised corneal integrity, secondary infections, prolonged ocular surface irritation due to chronic exposure to allergens, release of inflammatory mediators, e.g., histamine) Collagen-related diseases (rheumatoid arthritis, systemic lupus erythematosus, scleroderma, Ehlers–Danlos syndrome, osteogenesis imperfecta)	Corneal lymphangiogenesis involves an immune response consisting of antigen presentation and antigen-presenting cells in the surrounding lymph nodes favoring corneal graft rejection. This condition is associated with corneal graft rejection, inflammatory disorders, and ocular surface diseases. Excessive lymphatic vessel growth can contribute to immune responses and might factor in corneal transplant rejection and pathological corneal vessel formation

**Table 2:** Selected proangiogenic factors.

VEGF	A family of proteins fundamental in angiogenesis induction
Fibroblast growth factor (FGF)	A family of growth factors vital for regulating angiogenesis
Transforming growth factor-beta (TGF-β)	Involved in various cellular processes, including angiogenesis
Angiopoietins	A group of proteins that regulate angiogenesis by interacting with cell receptors
Platelet-derived growth factor (PDGF)	Stimulates cell growth and differentiation, including vascular endothelial cells
Matrix metalloproteinases (MMPs)	Enzymes that degrade the ECM, which facilitates cell movement and is essential for new vessel formation
Insulin	Affects angiogenesis through various mechanisms, including regulating vascular growth factors
Proinflammatory cytokines	Cytokines secreted during inflammatory processes, such as interleukin-1 (IL-1) and tumor necrosis factor alpha (TNF-α), stimulate angiogenesis
Interleukins (e.g., IL-8)	Act as chemotactic factors for endothelial cells, promoting angiogenesis
Netrins	A family of proteins involved in developmental processes, including regulating blood vessel growth
Placental growth factor (PlGF)	Similar to VEGF. VEGF and PlGF act synergistically in regulating angiogenesis
Angiotensin II	A peptide that affects the angiogenesis processes, especially in the renin–angiotensin system
Chemokines	Some chemokines, such as CXCL12 (stromal cell-derived factor-1, SDF-1), play a role in attracting endothelial cells and promoting angiogenesis
TNF-α	Proinflammatory cytokine that stimulates angiogenesis in response to inflammation
Hypoxia-inducible factor (HIF)	A transcription factor that activates genes associated with the hypoxia response, including proangiogenic factors
Basic fibroblast growth factor (bFGF)	A form of FGF involved in regulating angiogenesis
Platelet-derived growth factor-BB (PDGF-BB)	A PDGF isomer that might be involved in angiogenesis processes
Insulin-like growth factor (IGF)	A family of proteins that affect many cellular processes, including angiogenesis
Angiotensinogen	An angiotensin precursor that affects angiogenesis via the renin–angiotensin system
Prostacyclin (PGI2)	A prostaglandin group eicosanoid that affects endothelial function and angiogenesis processes
Nicotinic acid	A substance that affects angiogenesis processes through various mechanisms
Growth differentiation factor-15 (GDF-15)	Growth factor of the TGF-β family growth factor that regulates angiogenesis
Connective tissue growth factor (CTGF)	Protein associated with the regulation of cell proliferation and ECM production processes, which can affect angiogenesis
Stromal cell-derived factor-1 (SDF-1)	The chemokine growth factor involved in cell migration and attraction processes. It also affects angiogenesis

**Table 3:** Selected antiangiogenic factors.

Angiostatin	A proteolytic fragment of plasminogen that inhibits the growth of new blood vessels
Endostatin	A peptide derived from collagen XVIII, which inhibits angiogenesis
Thrombospondins	A cellular protein family that inhibits angiogenesis by interacting with growth factors and endothelial cells
Interferons	Interferons, especially interferon alpha and interferon beta, might exhibit antiangiogenic effects
Endoglin	Receptor-associated protein for TGF-β, which regulates angiogenesis
Pigment epithelium-derived factor (PEDF)	A factor derived from the pigment epithelium that exhibits antiangiogenic properties
Thrombomodulin	A protein present in the vascular endothelium that inhibits angiogenesis
Vasostatin	A VEGF-derived peptide that exhibits antiangiogenic activity
Short antiangiogenic peptides	Designed to inhibit angiogenesis, including short sequences derived from endostatin
Soluble VEGF receptors (sVEGFRs)	Act as traps to inhibit VEGF action
Small interfering RNA (siRNA) and antisense oligonucleotides	Block the expression of angiogenesis-related genes in a targeted manner
Canstatin	A collagen XVIII fragment exhibiting antiangiogenic properties
Tissue inhibitors of metalloproteinases (TIMPs)	Regulate the activity of metalloproteinases, enzymes involved in extracellular matrix degradation, which affects the angiogenesis process

**Table 4:** TGF-β inhibitors.

TGF-β receptor kinase inhibitors	Small-molecule inhibitors targeting the kinase domain of TGF-β receptors, such as SB431542 and LY2157299, are being investigated for their potential antifibrotic and anti-tumor effects
Anti-TGF-β antibodies: Fresolimumab	A monoclonal antibody that neutralizes all three TGF-β isoforms and has been studied in clinical trials for idiopathic pulmonary fibrosis and cancer
Anti-TGF-β antibodies: Trabedersen	An RNA-based drug designed to inhibit TGF-β2 production and that has been studied in clinical cancer trials
Ligand traps: Soluble TGF-β receptors	Fusion proteins or antibodies that act as decoy receptors sequestering TGF-β ligands and prevent them from binding to cell surface receptors
Smad inhibitors: SIS3 (Smad3 inhibitor)	A small-molecule inhibitor specifically targeting Smad3, a downstream signaling molecule in the TGF-β pathway
Natural compounds: Epigallocatechin gallate (EGCG)	Found in green tea, EGCG inhibits TGF-β signaling and has potential antifibrotic effects
Natural compounds: Curcumin	A compound found in turmeric that has been investigated for its ability to modulate TGF-β signaling in various diseases
Decorin	A proteoglycan that can bind to and neutralize TGF-β, preventing its interaction with its receptors
Pirfenidone	An indirect TGF-β inhibitor that is an antifibrotic drug that modulates TGF-β levels and signaling in fibrotic diseases

## Data Availability

Not applicable.
